# Automated Spin‐Assisted Layer‐by‐Layer Epitaxy Produces Highly Oriented Mixed‐Linker MOF Thin Films

**DOI:** 10.1002/anie.3901165

**Published:** 2026-09-10

**Authors:** Eleonora Afanasenko, Benedetta Marmiroli, Behnaz Abbasgholi Nejad Asbaghi, Barbara Sartori, Giovanni Birarda, Chiaramaria Stani, Matjaž Finšgar, Peter E. Hartmann, Mark Bieber, Emma Walitsch, Rolf Breinbauer, Simone Dal Zilio, Sumea Klokic, Heinz Amenitsch

**Affiliations:** ^1^ CERIC‐ERIC Trieste Italy; ^2^ Institute of Inorganic Chemistry University of Technology Graz Austria; ^3^ Istituto Officina dei Materiali – CNR‐IOM Trieste Italy; ^4^ Elettra Sincrotrone Trieste Trieste Italy; ^5^ Faculty of Chemistry and Chemical Engineering University of Maribor Maribor Slovenia; ^6^ Institute of Physical and Theoretical Chemistry University of Graz Graz Austria; ^7^ Institute of Organic Chemistry University of Technology Graz Austria

**Keywords:** crystal growth, liquid phase epitaxy, metal‐organic frameworks, orientation control, thin films

## Abstract

Control over crystallographic orientation in metal‐organic framework (MOF) thin films is crucial for exploiting their anisotropic properties in sensing, catalysis, and separation. Achieving reproducible, highly oriented films remains challenging, especially for mixed‐linker, pillared‐layered frameworks. Here we present an automated, spin‐assisted layer‐by‐layer liquid‐phase epitaxy (LbL‐LPE) strategy that enables rapid, ambient‐condition fabrication of highly oriented, mixed‐linker MOF thin films, demonstrated for Zn_2_BDC_2_DABCO (BDC = terephthalate, DABCO = 1,4‐diazabicyclo[2.2.2]octane). Correlative process monitoring by grazing‐incidence wide‐angle x‐ray scattering (GIWAXS), grazing‐angle infrared (GI‐IR) and UV‐Vis spectroscopy, contact‐angle measurements, scanning electron microscopy (SEM), and time‐of‐flight secondary ion mass spectrometry (ToF‐SIMS) ensures formation of uniform films with exceptional out‐of‐plane (001) orientation (degree of orientation > 85%, Hermans orientation parameter ∼0.95) and excellent reproducibility. This strategy enables high‐throughput fabrication of orientation‐controlled MOF thin films, providing a generalizable alternative to conventional LbL approaches. The resulting highly oriented Zn_2_(BDC)_2_(DABCO) thin films retain their crystallinity after prolonged exposure to 80% and short‐term exposure up to 90% relative humidity, whereas defect‐rich films undergo rapid degradation. Thus, this reproducible, automated strategy establishes the role of crystallographic orientation in humidity stability and advances the integration of oriented MOFs into optoelectronic, sensing, and membrane devices.

## Graphical abstract

An automated spin‐assisted layer‐by‐layer growth method enables rapid fabrication of highly oriented Zn_2_BDC_2_DABCO metal‐organic framework thin films under ambient conditions. Correlative characterization confirms excellent reproducibility, uniform film formation, and exceptional out‐of‐plane orientation exceeding 85%. The resulting films demonstrate remarkable humidity stability, retaining structural integrity under relative humidities up to 90%. These findings demonstrate that controlled MOF orientation provides a pathway toward improved functional performance of MOF thin‐film‐based systems, facilitating their integration into advanced sensing, separation, and optoelectronic devices.

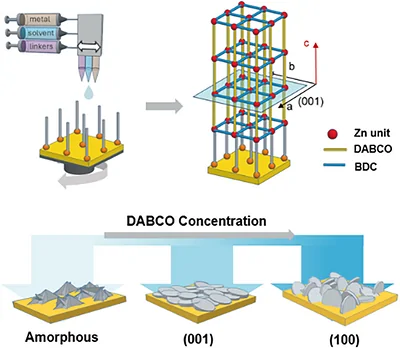

## 1 Introduction

The fabrication of oriented crystalline materials has become increasingly important for the development of films whose properties depend on alignment along a specific crystallographic axis [1, 2, 3]. A prominent example is given by metal‐organic frameworks (MOFs), consisting of ordered networks of metal nodes and organic linkers, which offer exceptional compositional tunability and high surface area and porosity, rendering them attractive for gas storage, sensing, and separation [4, 5, 6, 7]. For many of these applications, exact control over crystallographic orientation is essential because many MOF properties, including gas permeability [8, 9, 10], optical properties [11, 12], or photo‐induced structural dynamics [13, 14], exhibit directional dependence arising from lattice anisotropy. For instance, crystallographic orientation in MOF films enables optical birefringence and anisotropic fluorescence [11, 15], promotes enhanced directional gas permeation in membranes with preferential pore‐channel alignment [16], and governs structural dynamics in stimuli‐responsive frameworks [17, 18]. These examples highlight that crystallographic alignment can strongly enhance or suppress functional performance, making oriented MOF films critical for maximizing directional responses and enabling reliable integration into devices such as sensors, switches, and electronic components, where uniformity and reproducibility are required [19, 20, 21, 22, 23].

To date, several strategies have been developed to grow oriented MOF films, including liquid‐phase epitaxy (LPE) [24, 25], layer‐by‐layer assembly (LbL) [26], substrate‐seeded heteroepitaxy (SSH) [11, 27, 28], vapor‐assisted conversion (VAC) [29], and various external‐field‐assisted approaches, as comprehensively reviewed elsewhere [30, 31, 32]. Each method offers distinct advantages and inherent limitations, with only some enabling controllable or even tunable crystallographic orientation. A more detailed comparison among the fabrication methods is provided in Table S1. Within this context, layer‐by‐layer liquid‐phase epitaxy (LbL‐LPE) is particularly attractive due to its low chemical consumption and minimal solvent waste [24, 25, 33, 34]. Among LbL‐LPE approaches, the spin‐assisted method was introduced by Chernikova et al. [35]. particularly stands out for reduced solvent use, minimized cross‐contamination through direct deposition of reactants onto the substrate, precise thickness control via the number of deposition cycles, and shorter fabrication times compared to other LPE methods.

Despite these strengths, spin‐assisted LbL‐LPE has not yet been translated into a robust high‐throughput workflow that ensures reproducible orientation control and allows real‐time monitoring and adjustment of critical process parameters—capabilities that are crucial for transferability across different MOF chemistries [27, 36]. So far, only a limited number of studies have explored spin‐assisted LbL approaches for MOF film fabrication, primarily focusing on frameworks with a single ligand type, such as ZIF‐8, HKUST‐1, and Cu_2_(bdc)_2_·xH_2_O, SURMOF‐2‐type [37], or bimetallic MOF films [38]. In contrast, mixed‐ligand or pillar‐layered frameworks, highly relevant in the design of structurally adaptive materials, remain largely unexplored [6, 8]. In these systems, orientation control is particularly important for anisotropic pore contraction [11] and light‐responsive behavior [6, 9], when guest‐induced deformations [7] can propagate differently along distinct crystallographic directions, ultimately affecting the macroscopic functionality of the film [8].

Here, we address these challenges by developing an automated, spin‐assisted LbL‐LPE protocol with integrated monitoring of all critical processing steps, enabling the reproducible fabrication of homogeneous and highly oriented MOF films (Figure 1). The workflow incorporates contact‐angle analysis to verify self‐assembled monolayer (SAM) functionalization and UV‐visible spectroscopy to accurately adjust reactant concentrations. The structural quality and orientation of the resulting films are assessed through a correlative post‐synthetic characterization strategy combining grazing‐incidence wide‐angle x‐ray scattering (GIWAXS), grazing‐incidence reflectance (GI‐IR) spectromicroscopy, scanning electron microscopy (SEM), and time‐of‐flight secondary‐ion mass spectrometry (ToF‐SIMS). As a proof‐of‐concept system, we employ the flexible pillar‐layered framework Zn_2_BDC_2_DABCO (BDC = terephthalate, DABCO = 1,4‐diazabicyclo[2.2.2]octane), chosen for its structural flexibility and previously demonstrated exceptional photoresponsive behavior and efficient CO_2_ uptake in SSH‐grown oriented films as reported by some of the authors present herein [4, 13, 39].

**FIGURE 1:**
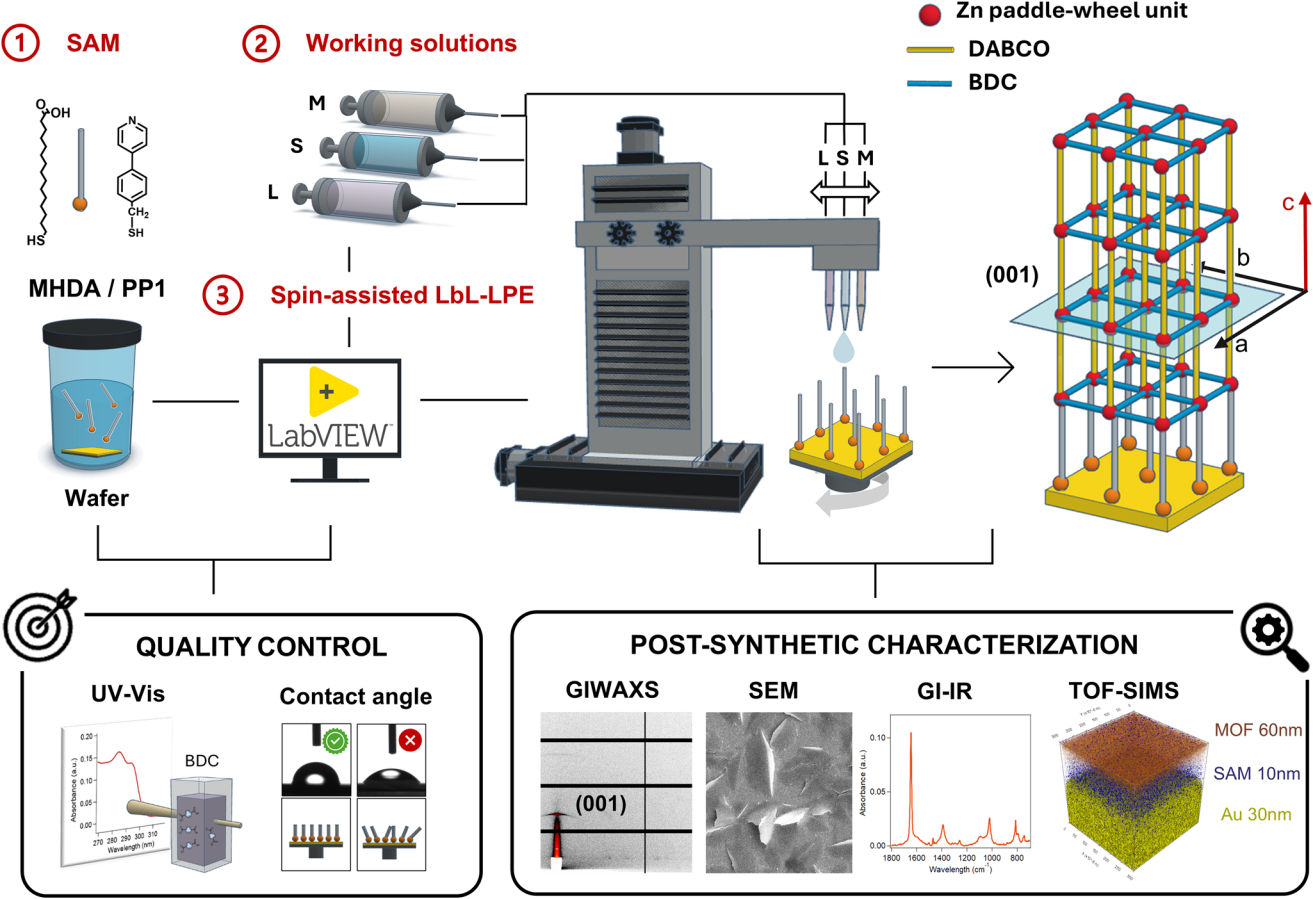
Schematic representation of the spin‐assisted LbL‐LPE protocol. The workflow comprises three key processing steps, each paired with quality control or (post‐synthetic) characterization: (1) gold wafers functionalization with MHDA or PP1 SAMs, with surface quality verified by contact angle measurements; (2) preparation of precursor solutions, with concentrations confirmed by UV‐Vis spectroscopy; (3) fully automated layer‐by‐layer deposition controlled via LabVIEW, followed by structural characterization of the resulting thin films. The illustration represents the structure viewed along the (001) direction.

In the automated spin‐assisted LbL‐LPE process, individual deposition and rinsing steps are completed within seconds, enabling rapid film growth under ambient conditions with low reagent consumption. A modular software package enables automated execution and logging of each fabrication step, ensuring precise process control and facilitating implementation. By systematically varying the metal (Zn) to linker (BDC:DABCO) ratios, we identify a regime that yields highly oriented films with the (001) reflection aligned parallel to the substrate. Deviations from this ratio lead to the emergence of partial (100) domains, as observed by GIWAXS and confirmed by SEM, highlighting the sensitivity of pillar‐layered frameworks to reactant stoichiometry. Finally, the reproducible growth of homogeneous Zn_2_BDC_2_DABCO films is achieved using both commercially available 16‐mercaptohexadecanoic acid (MHDA) and the synthesized pyridine‐terminated (4‐(4‐pyridyl)phenyl)methanethiol (PP1) [40].

The herein reported software‐controlled spin‐assisted LbL‐LPE platform enables the reproducible fabrication of homogeneous, highly oriented MOF thin films, including complex pillar‐layered and structurally adaptive frameworks. By combining precise crystallographic control with rapid, scalable deposition, this approach provides a reliable route to device‐compatible MOF films and facilitates access to orientation‐dependent properties for applications in gas separation, anisotropic sensing, optoelectronics, and stimuli‐responsive actuation.

## 2 Results and Discussion

### 2.1 Synthesis of the Zn_2_(BDC)_2_DABCO via LbL‐LPE Spin‐Coating

#### 2.1.1 LPE Spin‐Coating Protocol

The automated LPE spin‐coating protocol comprises three sequential steps, schematically outlined in Figure 1, each followed by a quality control check to ensure reproducible Zn_2_BDC_2_DABCO thin film growth with controlled orientation, thickness, and uniformity.

The deposition process was carried out on gold substrates, pre‐functionalized with self‐assembled monolayers (SAMs), which provide the initial surface for MOF film growth. The spin‐coating step is fully automated using a motorized arm equipped with three PTFE tubes, each connected to a separate syringe containing one of the working solutions: zinc acetate (M), the ethanolic mixed‐linker BDC‐DABCO solution (L), and ethanol as the washing solvent (S) (Figure S1a). The syringes are mounted on syringe pumps, which deliver the working solutions dropwise onto the substrate through the tubing (Figure S1b).

A single deposition cycle follows the sequence S–M–S–L–S and defines one growth cycle (Figure S1c). Zn_2_BDC_2_DABCO thin films were prepared using either 60 or 120 deposition cycles, with the number of repetitions and solution volumes fully programmable through LabVIEW‐controlled automation (National Instruments, USA). The tube‐to‐substrate distance was optimized to achieve uniform coverage, and the resulting films were dried under a gentle nitrogen flow immediately following the last deposition cycle. This fully programmable setup allows flexible tuning of all critical parameters to achieve reproducible, high‐quality MOF films. The specific optimization steps are discussed in detail in the following section.

### 2.2 Optimization of the LbL‐LPE Workflow

To ensure reproducibility and efficacy of the automated fabrication protocol, all parameters were carefully optimized, and strict monitoring of critical steps, defined above as the quality control, was implemented after each process step (Figure 1, steps 1–3). These key steps include, firstly, the functionalization of gold wafers with MHDA or PP1 SAMs, with surface quality verified by contact angle measurements following well‐established literature procedures [6]. Secondly, preparation of precursor solutions with concentrations confirmed by UV‐Vis spectroscopy, and, thirdly, the fully automated layer‐by‐layer deposition to grow oriented Zn_2_BDC_2_DABCO films that are subsequently characterized structurally.

During the first step in the fabrication process (Figure 1, step 1), the gold wafers were functionalized with MHDA or PP1 SAMs, which serve as the anchoring layer for subsequent MOF growth. Here, only freshly SAM‐functionalized substrates less than 48 h old were employed for the LPE spin‐coating process to ensure reproducible MOF thin film growth. The quality of the SAM was first confirmed by contact angle measurements (Figure S2a), serving as the initial process quality control step. Generally, both SAM‐forming molecules are known to be chemically stable across a broad range of experimental conditions and are widely used as anchoring layers in surface‐mounted MOFs [41, 42, 43]. However, prolonged storage of functionalized wafers is known to induce surface disorder, which can adversely affect subsequent MOF growth [44, 45]. Well‐ordered MHDA layers exhibit pronounced hydrophilicity due to their terminal –COOH groups, as reflected by the measured contact angles (*α*) of approximately *α* = 30°, while defective or disordered SAMs display increased hydrophobicity with contact angles around *α* = 50° (Figure S2a). A similar trend was observed for PP1 SAMs with *α* = 35° for well‐ordered layers; after the loss of order, the values increased to *α* = 53°, reflecting the combination of a hydrophilic pyridine moiety and a hydrophobic aromatic backbone. According to previous reports [44, 45, 46, 47, 48], SAM degradation is attributed to UV‐induced oxidation of the sulfur head groups and can be further promoted by both poor gold substrate quality and suboptimal functionalization procedures. Thus, for the process herein, only freshly prepared SAM‐functionalized substrates (< 48 h) were used to minimize surface disorder and ensure reproducible crystallographic orientation and related film properties (vide infra).

During the second step in the fabrication process (Figure 1, step 2), focused on the preparation of the working solutions of the linkers (H_2_BDC, DABCO) and metal (Zn(ac)_2_), the ethanolic H_2_BDC solution is first analyzed by UV‐Vis spectroscopy to verify its concentration. DABCO is added only afterward to the H_2_BDC solution, as the overlapping absorbance bands of both linkers preclude reliable differentiation in a single solution (Figure S2b). DABCO in the separate solution was not detected, as its characteristic absorption band lies in the 190 nm region, close to the lower detection limit of the instrument.

Working solutions were typically used for no more than 3 days to avoid ligand crystallization or the formation of Zn(OH)_2_ [49, 50]. It is important to note that one of the key factors influencing the quality of films produced by the LPE spin‐coating process is the careful optimization of both the linker‐to‐linker and metal‐to‐linker ratios, which will be discussed in detail in the following sections. For the conditions used here, the nominal concentrations yielding reproducibly oriented and uniform MOF films were determined with Zn:BDC:DABCO = 0.1 mM:0.1 mM:0.3 mM.

Only after successful validation of step 1 (SAM functionalization) and step 2 (working solution preparation) is the fully automated deposition process carried out (Figure 1, step 3). During this step, 33 µL of each solution, determined empirically, are dispensed sequentially onto the functionalized substrate at a flow rate of 0.2 µL min^−1^ while the substrate rotates continuously at 550 rpm. One layer is deposited within 40 s. The tube‐to‐substrate distance was fixed at 10 mm to control droplet impact and prevent coverage issues arising from the surface tension effect as visually inspected during process optimization: higher spinning speeds (800–1000 rpm) caused premature drying and prevented film growth, while lower speeds (450–500 rpm) led to unstable rotation and uneven coverage. Unlike conventional spin‐assisted approaches involving intermittent drying, the optimized rotation speed of 550 rpm maintains a persistent liquid layer on the substrate, enabling continuous, in‐solution MOF self‐assembly under precise stoichiometric control. Between each solution dispensing step, the sample is washed with ethanol to remove excess reagents and maintain uniform deposition. At completion of the deposition cycles, the film is removed from the spin‐coater and dried under a gentle nitrogen stream. Leaving the wafer to dry under continuous spinning after completion of the deposition step results in a loss of crystalline orientation, leading to more isotropic GIWAXS patterns (Figure S2c). In contrast, grazing‐incidence infrared (GI‐IR) spectromicroscopy confirmed that the chemical structure is preserved, as no differences are observed in the GI‐IR spectra (Figure S2d).

Following the optimized automated deposition conditions described above, the homogeneity and chemical composition of Zn_2_BDC_2_DABCO films were assessed using GI‐IR, which provides spatially resolved analysis across large substrate areas. To this aim, the entire wafer was raster‐scanned, collecting individual GI‐IR spectra distributed across the substrate. At a molar ratio of BDC:DABCO = 1:1:3, complete wafer coverage was achieved at the selected spinning speed without any edge inhomogeneities as commonly seen in spin‐coated films (Figure S3a). Also, other molar ratios were assessed by measuring dedicated areas in the center and on the sides (Figure S3b), showing good homogeneity at 60 cycles, which was further verified by measuring GI‐IR spectra of independently synthesized samples. The GIWAXS data further confirm that the grown MOF is homogeneous over the entire sample (Figure S3c). The reproducibility of the automated LbL‐LPE process was verified by quantifying crystallite orientation via the degree of orientation and Hermans orientation parameter, described in more detail in the following sections. Additional post‐synthetic characterization, including the impact of variations in reagent molar ratios on morphology and crystallinity, is presented in the following section.

### 2.3 Structural and Morphological Characterization of LbL‐LPE Spin‐Coated Films

Achieving reproducible, highly oriented MOF thin films under ambient conditions requires precise control over reactant stoichiometry and deposition parameters. This is because the quality and orientation of MOF thin films critically depend on the relative amounts of linker and metal precursors, as dictated by fundamental principles of reactant stoichiometry and the roles of limiting and excess reagents in MOF assembly [51, 52, 53, 54]. To identify the conditions that yield uniform, preferentially oriented films under the process conditions described in the previous chapter, we systematically varied the BDC:DABCO molar ratio (1:1, 1:2, 1:3, 1:4, 1:5) and the number of deposition cycles (60 or 120). The resulting films were characterized by SEM and GIWAXS to assess morphology and crystallographic orientation, while their chemical composition was analyzed using GI‐IR spectromicroscopy (Figure S4). GI‐IR peak assignments (Table S4) were guided by DFT‐calculated spectra, with computational details provided in the Supplementary Information (Chapter 5, Figures S5–S11, Tables S2–S4).

We further examined how the linker ratio influences framework formation and crystallographic orientation in Zn_2_BDC_2_DABCO thin films. GIWAXS measurements reveal a pronounced dependence of the crystallographic orientation on the BDC:DABCO ratio (Figure 2a). At a ratio of 1:1, MOF growth is suppressed on PP1‐functionalized substrates, as evidenced by weak and diffuse reflections, while films grown on MHDA‐functionalized substrates show no detectable diffraction. The GI‐IR spectroscopy confirms the limited framework formation, with a peak at 1585 cm^−^^1^ indicative of Zn‐BDC coordination, but the absence of the C–N–C bending vibration at 1469 cm^−^^1^ indicates insufficient DABCO incorporation (Figure 2b).

**FIGURE 2:**
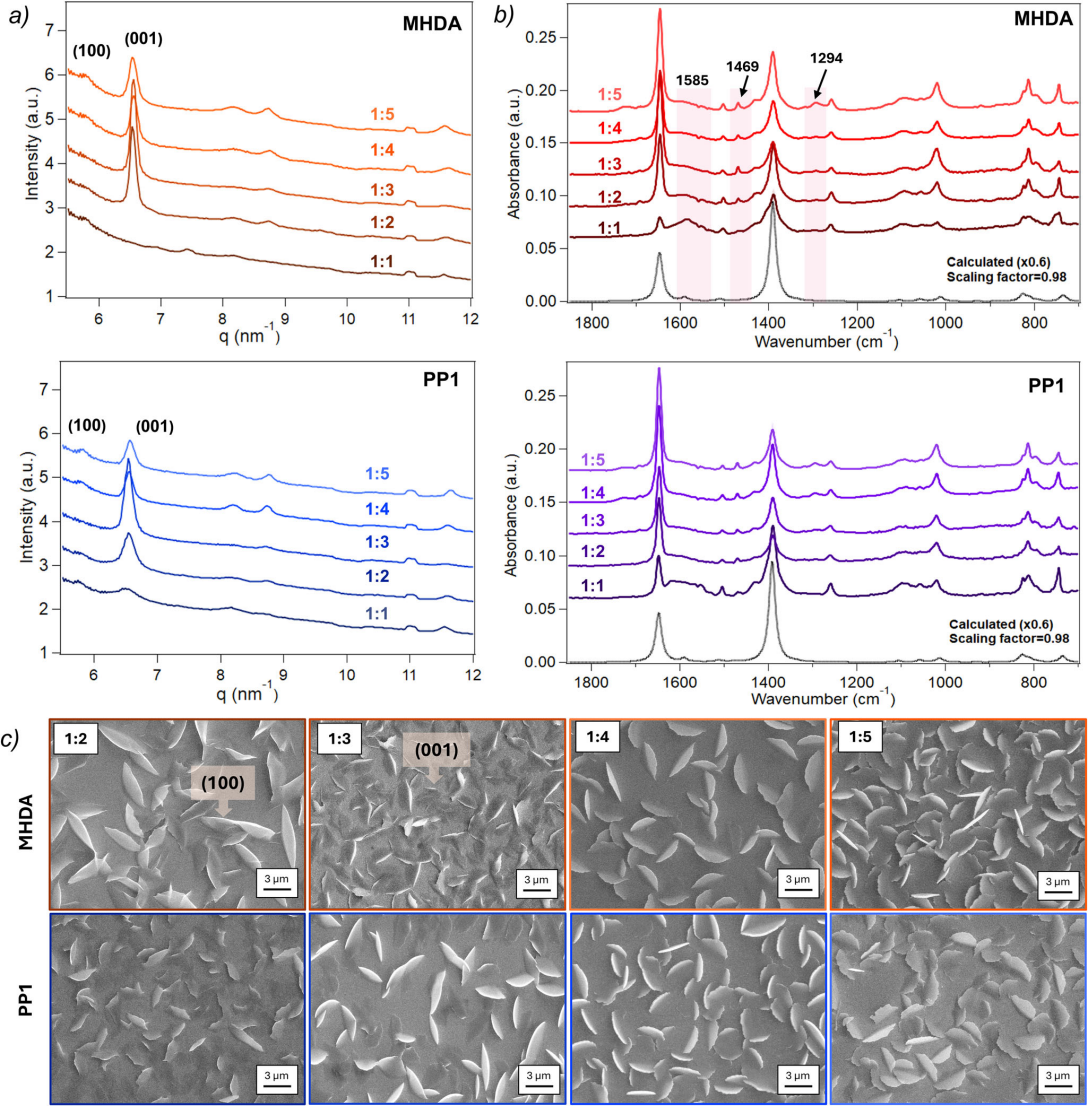
Characterization of Zn_2_BDC_2_DABCO thin films after 60 cycles grown on MHDA‐ and PP1‐functionalized substrates as a function of BDC:DABCO molar ratio (1:1, 1:2, 1:3, 1:4, 1:5). (a) Radially integrated GIWAXS patterns; (b) GI‐IR‐spectra of the films. The calculated spectrum is obtained from the V‐exp. model structure. The band at 1294 cm^−1^ corresponds to unreacted DABCO, while the band at 1469 cm^−1^ indicates coordinated DABCO. The band at 1585 cm^−1^ confirms coordination of Zn to BDC ligands. (c) SEM images of MOF films deposited on MHDA (top) and PP1 (bottom), for different BDC:DABCO ratios, indicating (001) aligned crystalline plates and (100) ones perpendicular to the surface.

Increasing the relative DABCO content enhances framework formation, reflected by a sharp increase in the intensity of the (001) reflection. Films grown after 60 deposition cycles at ratios above 1:1 display a strong diffraction peak of the (001) lattice plane, indicating preferential out‐of‐plane alignment of the MOF lattice (Figure S12a, for out‐of‐plane and in‐plane directions). This alignment occurs independently of SAM functionalization, demonstrating that stoichiometric control dominates over surface chemistry in determining lattice orientation along the surface normal (Figure 2a). Comparison with the powder diffraction pattern of the bulk structure confirms that the film system adopts the tetragonal *P4/mmm* structure (Figure S12) [55] GI‐IR spectromicroscopy further confirms coordination of the BDC linker, showing characteristic asymmetric and symmetric carboxylate stretching vibrations at 1624 and 1402 cm^−^^1^, respectively (Figure 2b).

Films grown at BDC:DABCO ratios of 1:2 and 1:3 exhibit the highest out‐of‐plane orientation and uniformity, with the 1:3 ratio approaching a nearly single‐crystal‐like alignment, as evidenced by diffraction intensity focused into discrete spots (Figure 2a). When the DABCO content is increased beyond 1:3 (ratios 1:4 and 1:5), the azimuthal intensity distribution of the (001) reflection broadens over the azimuthal angle (χ), reflecting reduced lattice alignment. The overall intensity of the (001) reflection decreases, indicating less well‐ordered films. This is confirmed by GI‐IR measurements, where spectra of the 1:5 ratio show a peak at 1294 cm^−1^ corresponding to unreacted DABCO. For all films with ratios above 1:1, the dominant out‐of‐plane (001) reflection indicates preferential alignment of the c‐axis perpendicular to the substrate. Notably, no evidence is observed for a well‐defined in‐plane registry, such as orthogonal alignment of (h00) and (0k0) planes across the surface, indicating that the films are uniaxially oriented only along the out‐of‐plane direction (Figure S12c–e).

To examine the morphology of the Zn_2_BDC_2_DABCO films and to correlate crystallite arrangement with the observed out‐of‐plane orientation, SEM imaging was performed (Figure 2c). At BDC:DABCO ratios of 1:2 and 1:3, the films comprise densely packed, plate‐like crystallites aligned parallel to the substrate, consistent with the dominant (001) out‐of‐plane orientation observed in GIWAXS (vide supra, Figure 2a). In contrast, films with higher DABCO content (1:4 and 1:5) display crystallites of similar shape and thickness but with a broader distribution of tilt angles relative to the substrate, giving rise to partial (100) crystallographic domains (Figure 2a). This mixed‐orientational growth behavior is similar to that previously reported in Cu_2_(F_4_BDC)_2_(DABCO) [41], where plate‐like (001)‐oriented structures were achieved by tuning the synthesis temperature. In that system, twinning within the paddle‐wheel nodes was proposed as a mechanism for deviations from perfect (001) orientation [41]. In the present Zn_2_BDC_2_DABCO films, a similar mechanism can rationalize the observations: at low DABCO content (1:2), insufficient coordination allows some Zn_2_ paddle‐wheel units in the upper layers to rotate around the σ‐bonds of the BDC linker. These rotated units act as nucleation sites, introducing distortions into initially (001)‐oriented crystallites, leading to twinning. At the optimal ratio of 1:3, this behavior is largely suppressed, with more than 85% of crystallites aligning along the (001) direction (vide infra). Conversely, excessive DABCO (1:4–1:5) competes with Zn^2+^ binding, which disrupts the formation of Zn‐BDC 2D sheets and promotes more isotropic crystal orientations, consistent with both SEM and GIWAXS observations (Figure 2a,c). These results are also consistent with previous observations in Cu_2_(BDC)_2_(DABCO) which is isostructural to the model system Zn_2_(BDC)_2_(DABCO) [56].

Increasing the number of deposition cycles to 120 further affects crystallographic orientation. A weak peak at *q* = 5.8 nm^−1^ emerges, indicative of (100)‐oriented domains (Figure 3a), whereas films grown with 60 cycles show no additional peaks (Figure 2a). This trend occurs irrespective of the BDC:DABCO ratio and likely arises from the cumulative effect of sequential layer growth on already twinned or distorted layers (Figure 3b). No significant differences are observed in the GI‐IR spectra of samples prepared with 120 cycles compared to those with 60 cycles, apart from an overall increase in peak intensity (Figure 3c), indicating that the chemical structure remains unchanged. These observations highlight that both stoichiometric balance and the number of deposition cycles are key parameters for achieving highly oriented, uniform MOF films with minimal defects.

**FIGURE 3:**
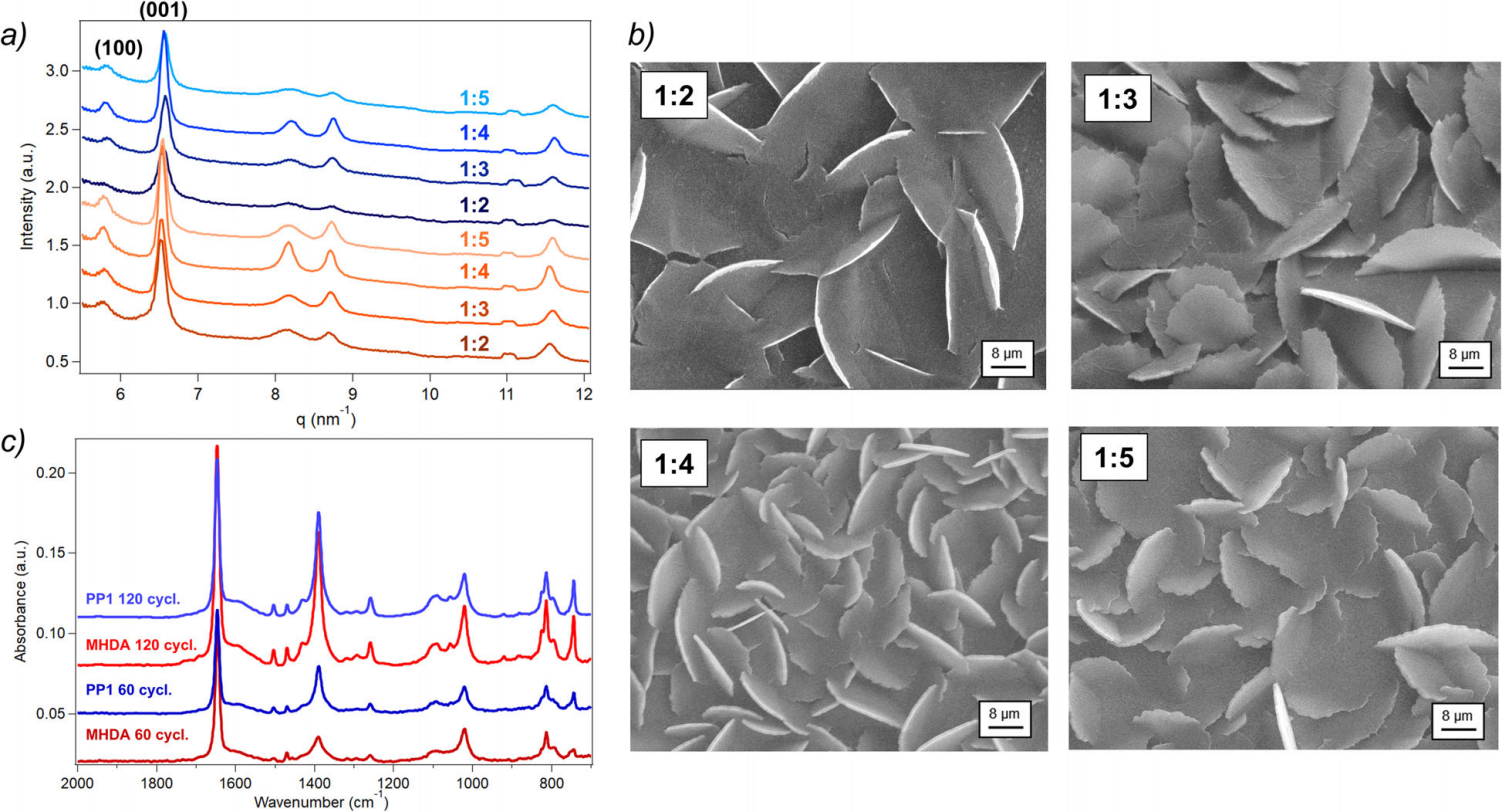
Characterization of Zn_2_BDC_2_DABCO thin films after 120 cycles. (a) Radially integrated GIWAXS patterns on MHDA (orange) and PP1 (blue) functionalized substrates; (b) SEM images of the films with different molar ratios; (c) GI‐IR spectra of the BDC:DABCO = 1:3 ratio.

Cross‐sectional SEM images prepared by FIB‐SEM (Figure S13a,b) show that both (001)‐ and (100)‐oriented crystallites have comparable thicknesses (∼64 nm) and grow directly on top of the SAM layer. Early‐stage (001)‐oriented crystals serve as seeding nodes for the subsequent growth of tilted and (100)‐oriented crystallites. EDS analysis further indicates that the regions between crystallites contain only minor metal (Figure S13b), suggesting that film formation proceeds via isolated nucleation sites rather than continuous layer growth, consistent with a Volmer‐Weber growth mechanism [15, 56, 57]. Additional SEM images of samples with an equimolar BDC:DABCO ratio confirm their amorphous structure (Figure S13c).

To further probe the film composition, ToF‐SIMS analysis was performed. Three‐dimensional reconstructions reveal NH^−^, C_6_H_5_^−^, Au_3_^−^, and S^−^ signals, corresponding to nitrogen‐containing fragments, aromatic moieties, the gold substrate, and sulfur species from the SAM, respectively (Figure 4a,b). Depth profiling using a gas cluster ion beam (GCIB) shows a largely uniform elemental distribution for films prepared at BDC:DABCO ratios of 1:3 and 1:5 (MHDA‐functionalized), with an increased NH^−^ signal in the 1:5 sample (Figure 4c) reflecting excess DABCO, in agreement with GI‐IR spectroscopy (Figure 2b). Sulfur‐containing species are localized at the gold/MOF interface, confirming the presence of the SAM, while Zn‐related signals co‐localize with aromatic (C_6_H_5_^−^) fragments, indicating homogeneous incorporation of Zn within the MOF framework. Two‐dimensional images of the ZnOH^−^ signal are homogeneously distributed across the scanned area, reflecting the presence of Zn–O(H) species within the film (Figure 4d). The formation of Zn(OH)_2_ is unlikely, as its characteristic signals would produce clear features in the GI‐IR spectrum of the fabricated films, which were not observed (at 3300 cm^−1^). Therefore, the ZnOH^−^ signal is more likely generated from ZnO via hydrogen attachment during Bi_3_^+^ bombardment or post‐ionization processes and should be considered a marker of Zn–O(H) species rather than a unique identifier of a single phase. The ZnOH^−^ coverage of the scanned area was calculated to be 50.6% for the sample with the molar ratio BDC:DABCO = 1:3 and 19.8% for the one with the ratio BDC:DABCO = 1:5. These results are consistent with the EDS analysis, which indicates that the crystals are spatially separated across the SEM‐imaged surface.

**FIGURE 4:**
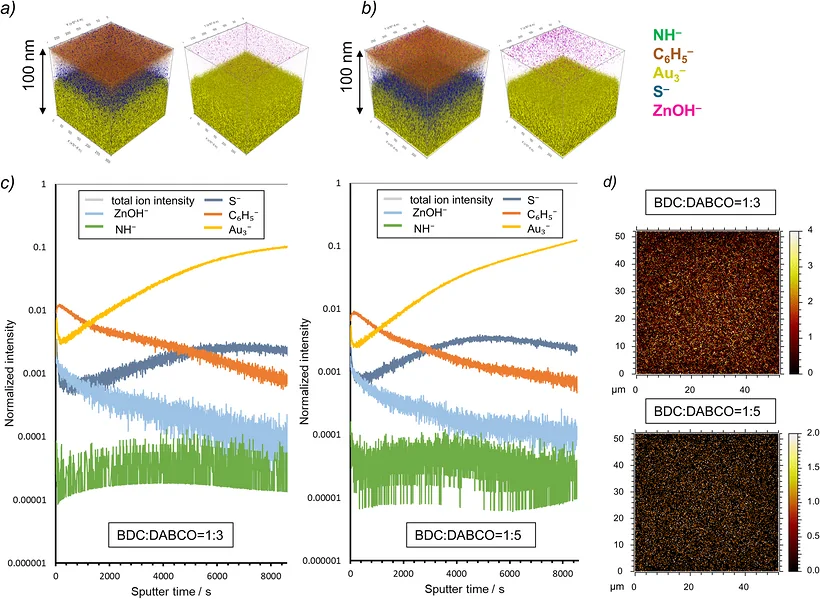
Characterization of Zn_2_BDC_2_DABCO thin films by ToF‐SIMS. Three‐dimensional ToF‐SIMS reconstructions of samples with the molar ratios Zn:BDC:DABCO = 1:1:3 (a) and 1:1:5 (b) on the MHDA self‐assembled monolayer (60 cycles), confirming the thickness measured from SEM images. (c) GCIB depth profiles showing the depth distribution of NH^−^, C_6_H_5_^−^, Au_3_^−^, S^−^, and ZnOH^−^ signals. (d) Two‐dimensional ToF‐SIMS images showing the distribution of the ZnOH^−^ signal on the MOF film surface (topmost position).

Film thickness was determined by 3D profilometry of sputtered craters (Figure S14), yielding a total thickness of 130 ± 15 nm for representative samples. This includes ∼50 nm of Au, ∼10 nm of SAM, and ∼70 nm of MOF, which is in good agreement with the crystallite dimensions observed by SEM.

Building on these observations, the influence of the metal‐to‐ligand ratio on film orientation was systematically investigated for samples grown for 60 deposition cycles on MHDA‐functionalized substrates (Figure 5). Increasing the metal concentration at a BDC:DABCO ratio of 1:1 did not result in crystal formation, consistent with the previously identified limitation of insufficient linker content (Figure 5a). At the optimal ligand ratio of 1:3, reducing the zinc acetate concentration to 0.05 mM yielded well‐oriented (001) films, albeit with incomplete substrate coverage, particularly at the sample edges, due to insufficient material deposition, as confirmed by SEM and GI‐IR spectroscopy (Figure 5b,c). Increasing the metal concentration to 0.2 mM resulted in uniform, highly oriented films, whereas further increases beyond 0.3 mM led to a loss of preferential orientation, as evidenced by the GIWAXS patterns (Figure 5a).

**FIGURE 5:**
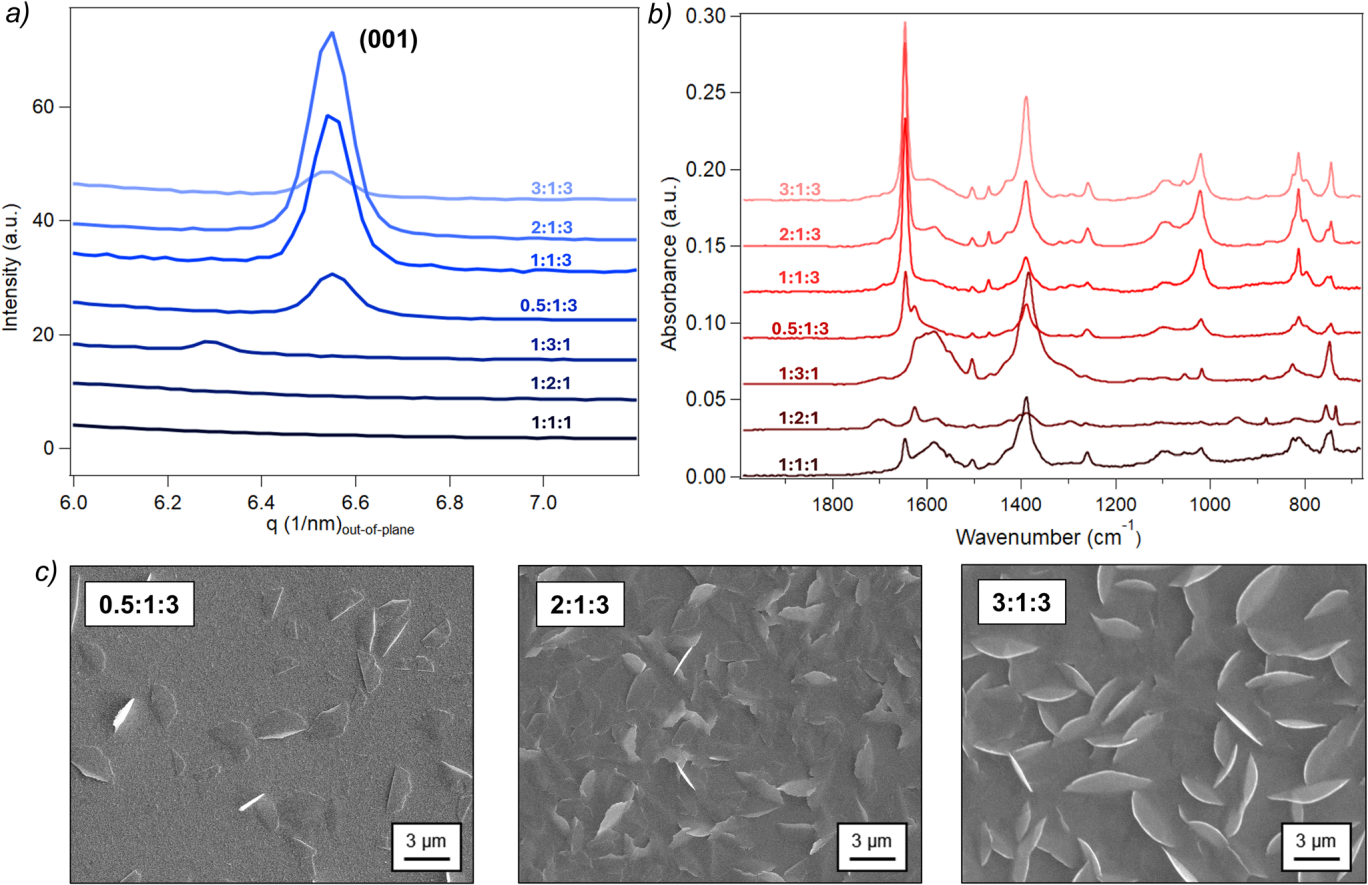
Characterization of samples prepared with 120 deposition cycles at varying Zn‐to‐ligand ratios on an MHDA self‐assembled monolayer: (a) GIWAXS, out‐of‐plane cut to compare (001) reflection, (b) GI‐IR, and (c) SEM data of the samples prepared by the variation of the Zn‐BDC‐DABCO molar ratios.

### 2.4 Quantitative Analysis of Film Quality and Reproducibility Evaluation

To quantify lattice alignment and assess reproducibility, the degree of orientation (DO) and Herman's orientation parameter (HOP) were evaluated from GIWAXS data. A detailed description of the analysis procedure is provided in the Supplementary Information (Chapter 10, Figure S15) [58, 59]. While both parameters describe crystallite orientation, they reflect complementary aspects: DO quantifies the fraction of aligned crystallites within the film with higher values indicating more homogeneous, well‐aligned films, whereas lower values point to increased misalignment or heterogeneity. HOP provides a quantitative measure of the degree of preferential orientation based on the azimuthal intensity distribution. HOP values range from 1 (perfectly aligned along the reference axis) to 0 (random orientation) and ‐0.5 (perfect perpendicular alignment). Both orientation parameters provide a clear measure of how the MOF lattice aligns with respect to the substrate, and, because GIWAXS probes a relatively large area, they also reflect the overall homogeneity of the films. When combined with peak‐width analysis (full width at half maximum, FWHM), they provide complementary information on film quality and enable evaluation of reproducibility across samples, capturing both macroscopic texture and nanoscale crystallite arrangement. Unlike one‐dimensional out‐of‐plane GIWAXS scans or SEM micrographs, this approach quantitatively distinguishes oriented from isotropically distributed crystallite populations. Despite appearing similar in conventional analyses, MHDA‐ and PP1‐functionalized films are differentiated by DO and HOP, which reveal subtle structural differences and are discussed in detail in the following.

The extracted HOP and DO mean values for MHDA‐ and PP1‐functionalized films are summarized in Figure 6a,b and Table S5. For films prepared at a BDC:DABCO ratio of 1:3 on MHDA‐functionalized substrates, excellent alignment is achieved with DO = 85% ± 10% (Figure S16) and HOP values approaching unity (0.93), accompanied by narrow FHWM values. This functionalization and reactant ratio marks the optimum conditions and indicates that most crystallites are oriented with their (001) planes parallel to the substrate. At lower ratios (1:2), both DO and HOP decrease and exhibit a larger spread among different samples, reflecting reduced alignment and poorer reproducibility. Increasing the DABCO content beyond the optimal ratio of 1:4 and 1:5 results only in minor changes of the DO and HOP values, but a pronounced broadening of the azimuthal distribution, indicating a wider spread of crystallite orientations and reduced alignment sharpness.

**FIGURE 6:**
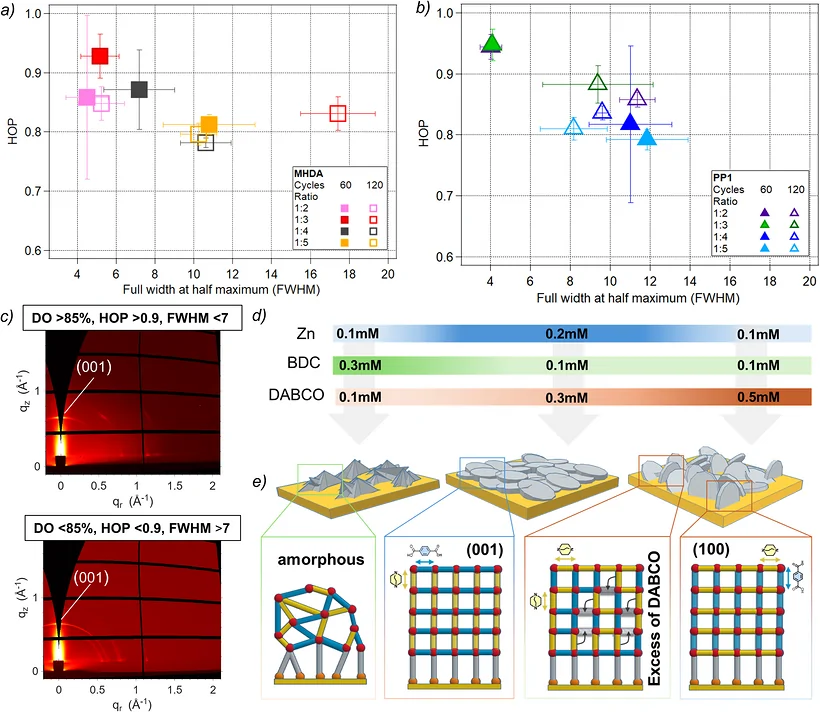
HOP versus full‐width at half maximum (FWHM) of the (001) reflection, where filled markers indicate 60 cycles and unfilled markers 120 cycles for (a) MHDA and (b) PP1 functionalized samples. The HOP and corresponding peak widths were averaged from triplicate samples (and from eight samples for the 1:3 ligand ratio at 60 cycles) under each synthetic condition. (c) Reshaped GIWAXS images showing highly oriented and isotropic scattering patterns. (d) Schematic representation of how changes in the molar ratio of reagents influence the crystalline orientation of LbL‐LPE‐synthesized films. (e) Illustration of different crystalline orientations and the mechanism of isotropic growth arising from the excess of DABCO ligand in the MOF structures on the substrate.

Despite similar GIWAXS patterns and SEM morphologies, quantitative analysis reveals subtle differences between SAM functionalizations: films grown on PP1‐functionalized substrates at a ratio of 1:3 exhibit lower DO values (61.30% ± 23.70%) and larger variability compared to MHDA‐functionalized films (88.64% ± 7.94%), indicating reduced reproducibility, while HOP values remain comparable (0.95). Increasing the number of deposition cycles to 120 for MHDA and PP1 films does not significantly affect the mean DO, while HOP decreases along with a clear increase in FWHM (Figure 6b), consistent with a higher fraction of tilted crystallites as previously inferred from SEM and GIWAXS analysis (vide supra, Figure 3). Based on the combined GIWAXS and spectroscopic data, three distinct alignment regimes can be identified for the herein fabricated Zn_2_BDC_2_DABCO films via the spin‐assisted automated LbL‐LPE process, schematically summarized in Figure 6d.

For BDC‐rich conditions, MOF growth is suppressed, where the limited structure formed is largely amorphous. Gradually increasing the DABCO concentration up to the optimal stoichiometry (BDC:DABCO = 1:3) produces highly oriented, uniform films dominated by (001)‐oriented crystallites with narrow azimuthal distributions (DO > 85%, HOP ≈ 0.9–1.0). In contrast, excess DABCO leads to broader orientation distributions and the emergence of mixed (001)/(100) domains, consistent with tilted crystallite growth observed by SEM. These regimes are reflected in the corresponding 2D GIWAXS patterns (Figure 6c), where sharp, localized reflections indicate high alignment, whereas azimuthally broadened intensity distributions signify increased disorder.

Overall, a BDC:DABCO ratio of 1:3 on MHDA‐functionalized substrates with 60 deposition cycles yields the most favorable combination of high orientation, uniformity, and reproducibility. Notably, the herein achieved DO (∼85%) compares beneficially with previously reported epitaxial Zn_2_BDC_2_DABCO films seeded either on Cu(BDC)_2_ (DO ∼ 45%) [11] or on Cu_2_BDC_2_DABCO (∼93%) [28]. This demonstrates that the spin‐assisted LbL‐LPE approach produces highly oriented MOF films. The ability to achieve well‐aligned films simply by adjusting the linker‐to‐linker and metal‐to‐linker ratios demonstrates that commercially available COOH‐terminated thiols can be used directly for LbL‐LPE growth of Zn_2_BDC_2_DABCO, eliminating the need for pyridine‐terminated SAMs, which require a lengthy synthesis and extensive purification (Supplementary information, chapter 1.3).

### 2.5 Hydrolytic Stability of Zn_2_(BDC)_2_(DABCO) Thin Films

One of the major challenges associated with many paddle‐wheel MOFs is their susceptibility to water‐vapor‐induced degradation, which restricts their deployment in applications such as gas storage, catalysis, and chemical sensing under humid operating conditions [60]. Improving the moisture stability of these frameworks is therefore an important prerequisite for their broader technological implementation. For bulk Zn_2_(BDC)_2_(DABCO) it has been reported that the framework undergoes collapse upon exposure to relative humidities above 60% [61]. This degradation proceeds through water molecules displacing the DABCO pillar ligands, resulting in the collapse of the three‐dimensional pillared framework into two‐dimensional Zn‐BDC layers [62]. Both experimental and computational studies have shown that this degradation pathway is accelerated at defect sites within the framework [60, 63, 64].

Considering the pronounced humidity sensitivity of bulk Zn_2_(BDC)_2_(DABCO), the effect of crystallite orientation on the humidity‐induced degradation behavior of thin films prepared via the herein developed spin‐assisted LbL‐LPE fabrication process was investigated. Specifically, the structural stability and chemical characteristics of highly oriented and defect‐rich isotropic films were compared under humid conditions. Highly oriented films (DO > 85%, HOP ∼ 0.95) obtained with the optimized BDC:DABCO = 1:3 molar ratio and defect‐rich, isotropic films prepared with a BDC:DABCO = 1:5 (DO < 85%, HOP < 0.8) ratio were exposed to 80% relative humidity for 24 and 48 h and subsequently characterized by GI‐IR, GIWAXS, and SEM.

GI‐IR measurements reveal that the highly oriented MOF thin films retain their structural integrity after exposure to 80% relative humidity for 48 h, exhibiting only minor conversion to Zn‐BDC, as evidenced by the appearance of the characteristic band at 1589 cm^−1^ (Figure 7 and Figure S17a). A comparable water‐induced transformation from Cu_2_(BDC)_2_(DABCO) to Cu_2_(BDC)_2_ has been reported for oriented thin films, where the crystallographic alignment was retained during the humidity‐induced phase transformation [57]. GIWAXS measurements further reveal a partial loss of crystallinity together with the emergence of the additional peak at *q* = 6.3 nm^−1^ indicating the formation of the Zn‐BDC crystalline structure (Figure S17b) [37]. SEM images confirm that the overall film morphology is retained, showing only limited degradation after prolonged humidity exposure (Figure 7).

**FIGURE 7:**
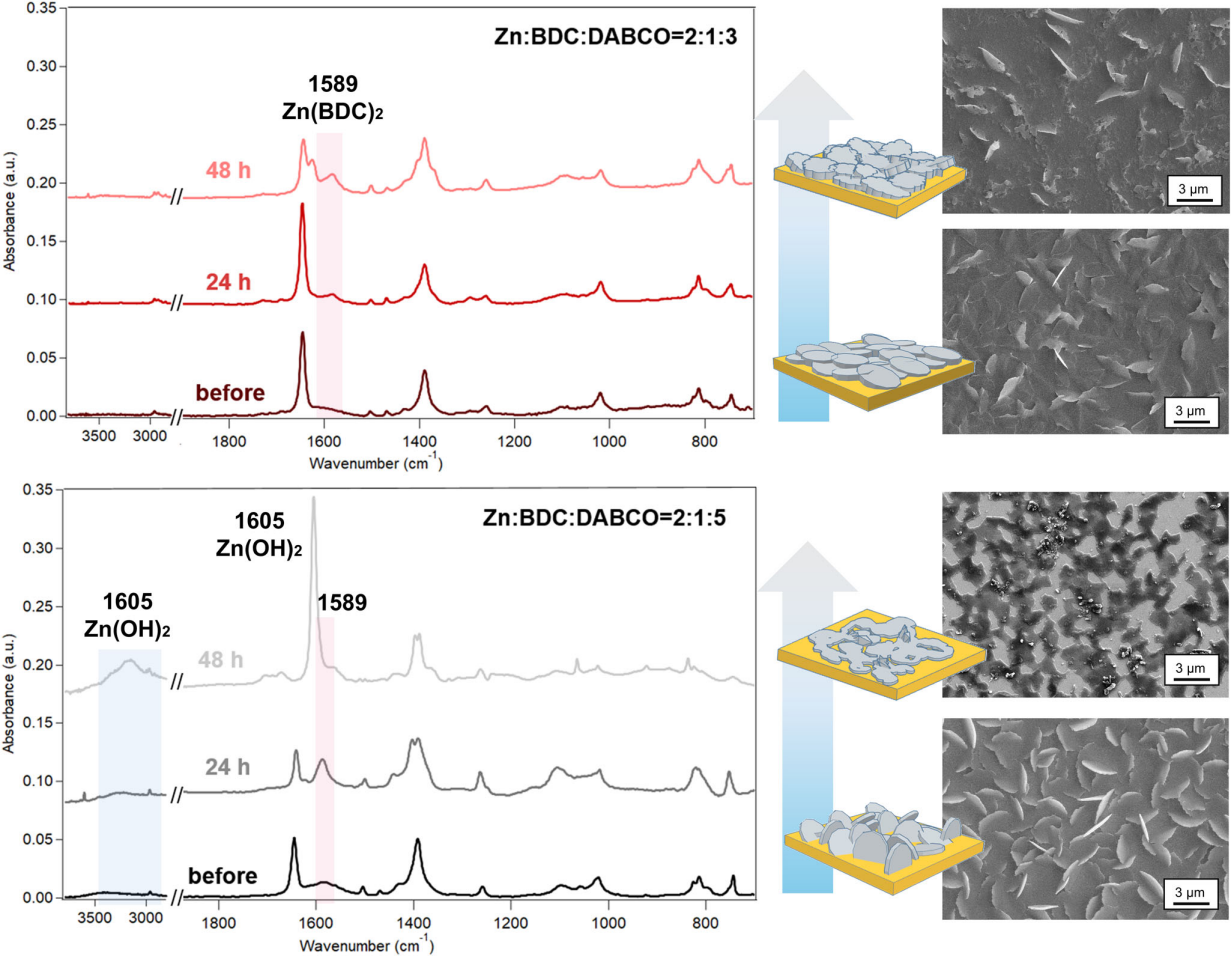
GI‐IR spectra of the highly oriented film with the molar ratio Zn:BDC:DABCO = 1:1:3 and the defect‐rich film with Zn:BDC:DABCO = 1:1:5 before and after the exposure to 80% relative humidity for 24 and 48 h together with a schematic illustration of the humidity‐induced transformations.

In contrast, the defect‐rich isotropic films prepared with a BDC:DABCO ratio of 1:5 exhibit substantially accelerated degradation. Partial conversion to Zn‐BDC is already observed after 24 h, followed by complete degradation into Zn(OH)_2_ after 48 h. This transformation is confirmed by the emergence of the characteristic band at 1605 cm^−1^ together with the broad hydroxyl absorption band at 3175 cm^−1^, characteristic of Zn(OH)_2_ [37]. The direct comparison between highly oriented and defect‐rich films demonstrates that the structural control achieved through LbL‐LPE directly translates into enhanced resistance against humidity‐induced degradation. Following humidity exposure, SEM micrographs reveal a complete disruption of the initial film morphology, resulting in an extensive degradation and transformation of the framework (Figure 7).

To further evaluate the structural response of the oriented films under dynamic humidity conditions, in‐situ GIWAXS measurements were performed under relative humidity up to 90%, employing humidity ramps of 1%, 2%, and 10%/min, with an equilibration time of one h at maximum humidity (Figure S18). The time‐resolved GIWAXS data show a shift of the reflection corresponding to the (001)‐oriented crystallites toward smaller *q* values, consistent with pore expansion upon water adsorption (Figure S18a). Importantly, this structural response is fully reversible for all humidity ramps, as evidenced by the complete recovery of the diffraction peak position upon returning to dry conditions (< 5% RH), together with the preservation of the original diffraction features. These results demonstrate that the highly oriented films undergo reversible water‐induced structural adaptation while maintaining their crystallinity and avoiding irreversible framework degradation, even under extreme humidity conditions. Overall, these results demonstrate that the highly oriented Zn_2_(BDC)_2_(DABCO) thin films exhibit significantly enhanced humidity tolerance compared with the bulk material [61]. Whereas the bulk framework readily degrades at 60% of relative humidity, the oriented thin films maintain their structural integrity after prolonged exposure to 80% relative humidity and display reversible structural responses even under short‐term humidity conditions up to 90%. These findings highlight that crystallite orientation is an important parameter for controlling moisture‐induced degradation and demonstrate that the structural control achieved through LbL‐LPE provides a pathway toward the fabrication of water‐vapor‐resistant MOF thin films.

## 3 Conclusion

This work demonstrates the fully automated fabrication of mixed‐linker, pillared‐layered MOF thin films via spin‐assisted LbL‐LPE under ambient conditions. Using Zn_2_BDC_2_DABCO as a model system, we show that precise control over linker‐to‐linker and metal‐to‐linker ratios enables the reproducible growth of homogeneous and highly oriented films without elevated temperatures or toxic solvents, supporting sustainable and low‐consumption film formation. The automated LbL‐LPE workflow provides fine control over key processing parameters, including SAM quality, reactant concentrations, deposition volume, and rotational speed, thereby enabling reliable tuning of film orientation and morphology. Correlative characterization by GIWAXS, GI‐IR, and SEM confirms exceptional alignment along the surface normal together with homogeneous substrate coverage. An optimal Zn:BDC:DABCO ratio of 1:1:3 yields uniformly covered films with strong out‐of‐plane (001) alignment (DO > 85%, HOP ∼ 0.95).

In contrast to the reported collapse of bulk Zn_2_(BDC)_2_(DABCO) above 60% relative humidity, the optimized thin films exhibit remarkably enhanced humidity tolerance. The high degree of crystallographic orientation achieved through LbL‐LPE significantly slows water‐vapor‐induced degradation, allowing the films to preserve their crystallinity after prolonged exposure to 80% relative humidity while undergoing fully reversible pore expansion under short‐term exposure to relative humidities up to 90%. Conversely, defect‐rich isotropic films undergo rapid and irreversible hydrolytic degradation, demonstrating that the structural control achieved through LbL‐LPE extends beyond morphology and translates directly into improved water‐vapor stability.

Beyond this model system, the results demonstrate that spin‐assisted LbL‐LPE is applicable to the fabrication of oriented MOF thin films, including structurally complex mixed‐linker architectures. The combination of automation, ambient processing, and low reagent consumption addresses key challenges in reproducibility and sustainable manufacturing of MOF thin films across laboratories. The high degree of crystallographic orientation is particularly relevant for orientation‐dependent applications, such as membranes, sensors, and optoelectronic devices. Overall, this work establishes a strategy for the controlled fabrication of highly ordered MOF thin films, aiming to facilitate their integration into functional device architectures.

## Author Contributions

**Eleonora Afanasenko**: conceptualization, investigation, formal analysis, funding acquisition, visualization, writing – original draft, writing – review and editing. **Benedetta Marmiroli**: data curation, investigation, validation, formal analysis. **Behnaz Abbasgholi‐N. A**.: methodology, investigation, visualization. **Barbara Sartori**: methodology, data curation, validation, formal analysis, writing – review and editing. **Giovanni Birarda**: software, methodology, data curation, formal analysis, resources. **Chiaramaria Stani**: data curation, validation, formal analysis, resources. **Matjaž Finšgar**: methodology, investigation, funding acquisition, visualization, resources. **Peter E. Hartmann**: software, investigation, formal analysis, writing – review and editing. **Mark Bieber**: methodology, investigation, formal analysis. **Emma Walitsch**: investigation, formal analysis. **Rolf Breinbauer**: methodology, data curation, validation, supervision, resources. **Simone Dal Zilio**: methodology, software, validation, resources. **Sumea Klokic**: conceptualization, investigation, validation, formal analysis, supervision, funding acquisition, project administration, writing – original draft, writing – review and editing. **Heinz Amenitsch**: conceptualization, data curation, supervision, funding acquisition, project administration, resources, writing – review and editing.

## Conflicts of Interest

The authors declare no conflicts of interest.

## Supporting information




**Supporting File**: anie74661‐sup‐0001‐SuppMat.docx.

## Data Availability

The data that supports the findings of this study are available in the supplementary material of this article.
