# Supplementary material for: Automated Spin‐Assisted Layer‐by‐Layer Epitaxy Produces Highly Oriented Mixed‐Linker MOF Thin Films
Source: Angew Chem Int Ed Engl. 2026 Sep 10;65(44):e3901165. doi: 10.1002/anie.3901165 (PMC13703917; doi:10.1002/anie.3901165)
Supplement: Supplementary file 1 [file ANIE-65-e3901165-s001.docx]

**Automated Spin-Assisted Layer-by-Layer Epitaxy Produces Highly Oriented Mixed-Linker MOF Thin Films**

**SUPPLEMENTARY INFORMATION**

Eleonora Afanasenko,^1^ Benedetta Marmiroli,^2^ Behnaz Abbasgholi-NA,^3^ Barbara Sartori^2^, Giovanni Birarda,^4^ Chiaramaria Stani,^4^ Matjaž Finšgar,^5^ Peter E. Hartmann,^6^ Mark Bieber,^7^ Emma Walitsch,^7^ Rolf Breinbauer,^7^ Simone Dal Zilio,^3^ Sumea Klokic^2*^ and Heinz Amenitsch^2*^

1 CERIC-ERIC, Trieste, Italy
2 Institute of Inorganic Chemistry, University of Technology, Graz, Austria
3 Istituto Officina dei Materiali - CNR-IOM, Trieste, Italy
4 Elettra Sincrotrone Trieste, Trieste, Italy
5 Faculty of chemistry and chemical engineering, University of Maribor, Slovenia
6 Institute of Physical and Theoretical Chemistry University of Graz, Austria
7 Institute of Organic Chemistry, University of Technology, Graz, Austria

**Table of contents**

[1.	Comparative Analysis of MOF Thin-Film Fabrication Methods	3](#_Toc236214481)

[2.	Experiment	4](#_Toc236214482)

[2.1.	Materials and methods	4](#_Toc236214483)

[2.2.	Synthesis of the Zn₂BDC₂DABCO thin films	4](#_Toc236214484)

[2.3.	Synthesis of the 4-(4-pyridyl)phenyl)methanethiol (PP1)	5](#_Toc236214485)

[2.4.	Characterization techniques	9](#_Toc236214486)

[2.4.1.	Grazing Incidence Small Angle X-Ray Scattering (GIWAXS) Experiments	9](#_Toc236214487)

[2.4.2.	IR spectromicroscopy	10](#_Toc236214488)

[2.4.3.	SEM and EDS measurements	10](#_Toc236214489)

[2.4.4.	ToF-SIMS measurements	10](#_Toc236214490)

[2.4.5.	3D Profilometry	11](#_Toc236214491)

[3.	LPE Spin-Coating Workflow	12](#_Toc236214492)

[4.	Assessment of film uniformity and fabrication reproducibility	14](#_Toc236214493)

[5.	DFT calculations	16](#_Toc236214494)

[5.1.	Computational Details	16](#_Toc236214495)

[5.2.	Detailed Information on the Computations	17](#_Toc236214496)

[6.	Calculated vs. experimental IR spectra and vibrational peaks assignment	25](#_Toc236214497)

[7.	GIWAXS Characterization of Zn₂(BDC)₂(DABCO) Films	27](#_Toc236214498)

[8.	Surface and cross-section morphology	29](#_Toc236214499)

[9.	Thin film thickness measurement with 3D profilometry	30](#_Toc236214500)

[10.	Degree of orientation and Hermans orientation parameter calculations	31](#_Toc236214501)

[11.	Hydrolytic Stability of Zn_2_(BDC)_2_(DABCO) Thin Films	35](#_Toc236214502)

[References	37](#_Toc236214503)

# 1. **Comparative Analysis of MOF Thin-Film Fabrication Methods**

Table S1. Summary of representative MOF thin-film fabrication methods reported in the literature.^[1–3]^ Reported values are representative and vary depending on the MOF chemistry; the table therefore provides a simplified comparison of representative mixed-ligand MOF thin films.

| **Method** | **Orientation control** | **Thickness control** | **Fabrication time** | **Solvent usage** | **Temperature** | **Mixed-ligand MOFs** |
| --- | --- | --- | --- | --- | --- | --- |
| **Hydro/ Solvothermal^[^**^3–5^**^]^** | Low-moderate | ~ μm^A^ | 3-48 h | High  *(50-200mL)* | Elevated  *(~100–150 °C)* | yes |
| **Vapor-assisted conversion (VAC)^[^**^6,7^**^]^** | High | ~ nm-μm^B^ | 1-48 h^[8,9]^ | Very low  *(<10mL)* | Elevated  *(80–150 °C)* | no |
| **Electrochemical^[^**^4,10^**^]^** | Low-moderate | ~ μm^C^ | 10 min-8 h^[11,12]^ | Low  *(~50mL)* | Ambient-mild  *(RT–80 °C)* | yes |
| **Substrate-seeded heteroepitaxy (SSH) ^[^**^13,14^**^]^** | Very high | ~ nm-μm^D^ | 10 min-100 h^[15–17]^ | High-moderate  *(10ml – 200ml)* | Ambient or elevated | yes |
| **Pump-Assisted / Microfluidic^[^**^18–20^**^]^** | High | ~ nm | 10 min-2 h | Very low  *(<10mL)* | Ambient or elevated | no |
| ***Layer-by-Layer methods (LbL)*** | | | | | | |
| **Dip-coating^[^**^21^**^]^** | High | ~ several nm/layer *(number of layers/cycles)* | 6-48 h^[22–24]^ | Very high  *(1-6 L)* | Ambient or elevated | yes |
| **Spray deposition^[^**^25,26^**^]^** | Good |  | 10 min-1 h | Low-moderate  *(~50-100mL)* | Ambient | no |
| **Spin-assisted,** developed herein^[27]^ | High |  | 50 min | Very low  *(<10mL)* | Ambient | yes |

***^A^*** *Temperature, pressure, and growth time, precursor concentration*

*^B^ Single-layer growth, sequential injection of precursors (CVD), precursor density*

*^C^ Low temperature, electric field gradient, supporting electrolyte*

*^D^ Growth time*

# 2. **Experiment**

## 2.1. **Materials and methods**

Reagents and solvents are available commercially and were used as received without any further purification. The linkers benzene-1,4-dicarboxylic acid (BDC), 1,4-Diazabicyclo[2.2.2]octane (DABCO) as well as the 16-Mercaptohexadecanoic acid for the SAM were purchased from TCI Chemicals, while zinc acetate dihydrate was obtained from TCI Chemicals, absolute ethanol (EtOH, 99.8%) was bought from VWR Chemicals. Gold-deposited substrates (polycrystalline gold with thickness on Si substrate precoated with titanium, roughness < 1nm) were purchased from Georg Albert PVD-Beschichtungen. The spin-coating step was performed using a Laurell spinner (Model WS-650MZ-23NPPB), Norm-Ject 20 mL plastic syringes with a standard diameter of 19.4 mm, and programmable syringe pumps (Harvard PHD 4400). Experiments were performed at ambient conditions.

## 2.2. **Synthesis of Zn₂BDC₂DABCO thin films**

*Self-assembled monolayers* (SAMs) were formed on gold substrates sized 1x1cm by immersion in an ethanolic (absolute) 0.02 mM solution of 16-mercaptohexadecanoic acid (MHDA) containing 10 vol% acetic acid, or in a 0.02 mM solution of (4-(4-pyridyl)phenyl)methanethiol (PP1), for 24 hours.^[28]^ Prior to functionalization, the wafers were sonicated in absolute ethanol for 30 minutes to ensure thorough cleaning.

The linker working solution was prepared by dissolving 0.01 mmol of benzene-1,4-dicarboxylic acid (BDC) in 100 mL of ethanol and sonicating for 2 hours to ensure complete dissolution. After confirming the proper BDC concentration *via* UV-Vis spectroscopy, 0.03 mmol of 1,4-diazabicyclo[2.2.2]octane (DABCO) was added, and the solution was sonicated for an additional hour. The working metal solution was prepared by dissolving 0.01 mmol of Zn(CH_3_CO_2_)_2_·2H_2_O in 100 mL of ethanol, followed by one hour of sonication.

## 2.3. **Synthesis of the 4-(4-pyridyl)phenyl)methanethiol (PP1)**

1) **(4-Bromophenyl)methanethiol**

17.0 g 4-bromobenzylbromide (68.0 mmol, 1 equiv.) were dissolved in MeOH (150 mL). Afterwards, 8.55 g potassium thioacetate (74.8 mmol, 1.1 equiv.) were added and the resulting suspension was stirred at r.t. for 30 min in an argon-flushed three-necked round-bottom flask, equipped with a Teflon-coated magnetic stirring bar. After TLC and GC-MS indicated full conversion, 9.41 g K_2_CO_3_ (68.1 mmol, 1 equiv.) were added and the suspension was stirred for another 40 min at r.t. After complete consumption of the intermediate thioacetate (reaction monitoring via TLC and GC-MS), the reaction mixture was carefully acidified (pH = 6) with 1M aq. HCl and extracted with DCM (3 x 120 mL). The combined organic phases were dried over Na_2_SO_4_, filtered and concentrated under reduced pressure. The crude product was used in the next step without further purification.

**Yield**: 13.8 g (quant.), orange oil, C_7_H_7_BrS [203.10 g/mol].

**TLC:** R_f_ = 0.75 (cyclohexane:EtOAc = 9:1; UV).

**GC-MS**: (EI, 70 eV; MT_50_S): t_R_ = 5.22 min; *m/z* (%): 204 (26), 169 (100), 121 (6), 90 (30), 63 (12).

**^1^H NMR** (300 MHz, CDCl_3_): δ 7.44 (d, *J* = 8.3 Hz, 2H), 7.20 (d, *J* = 8.3 Hz, 2H), 3.69 (d, *J* = 7.6 Hz, 2H), 1.76 (t, *J* = 7.6 Hz, 1H; -S**H**) ppm.

**^13^C NMR** (76 MHz, CDCl_3_): δ 140.2, 131.9, 129.9, 121.0, 28.5 ppm.

2) **((4-Bromobenzyl)thio)triisopropylsilane**

An argon-flushed Schlenk flask, equipped with a Teflon-coated magnetic stirring bar, was charged with 11.6 g (4-bromophenyl)methanethiol (57.1 mmol, 1 equiv.) and dissolved in abs. THF (110 mL). The reaction mixture was cooled to 0 °C and 2.51 g NaH (62.8 mmol, 1.1 equiv.) were added in portions over 15 min. The Schlenk flask was equipped with a bubbler and the reaction mixture was stirred until the evolution of gas had ceased (50 min). Afterwards, 13 mL TIPSCl (62.8 mmol, 1.1 equiv.) were added dropwise over 15 min and the reaction mixture was stirred for 70 min at r.t. (reaction monitoring via GC-MS). Subsequently, the reaction was quenched by the addition of H_2_O (110 mL). The phases were separated and the aqueous phase was back-extracted with EtOAc (3 x 110 mL). The combined organic phases were dried over Na_2_SO_4_, filtered and concentrated under reduced pressure. The crude product was first purified via vacuum distillation (10.0 x 2.0 com Vigreux column, 0.041 mbar) and then via flash column chromatography (1 L SiO_2_, 26.0 x 8.0 cm, cyclohexane to cyclohexane:EtOAc = 10:1 (v/v)).

**Yield**: 13.5 g (66%), colourless liquid, C_16_H_27_BrSSi [359.44 g/mol].

**TLC:** R_f_ = 0.39 (cyclohexane; UV).

**b.p:** 119-120 °C (0.041 mbar).

**GC-MS**: (EI, 70 eV; MT_50_S): t_R_ = 7.69 min; *m/z* (%): 360 (1), 315 (50), 273 (7), 169 (100), 90 (24).

**^1^H NMR** (300 MHz, CDCl_3_): δ 7.42 (d, *J* = 8.3 Hz, 2H), 7.22 (d, *J* = 8.3 Hz, 2H), 3.69 (s, 2H), 1.30 (hept, *J* = 7.1 Hz, 3H), 1.13 (d, *J* = 7.1 Hz, 18H) ppm.

**^13^C NMR** (76 MHz, CDCl_3_): δ 140.0, 131.7, 130.3, 120.7, 29.7, 18.71, 12.9 ppm.

3) **4-(4-(((Triisopropylsilyl)thio)methyl)phenyl)pyridine**

An argon-flushed Schlenk flask, equipped with a Teflon-coated magnetic stirring bar, was charged with 1.67 g 4-pyridylboronic acid (90% purity, 12.2 mmol, 1.2 equiv.), 6.67 g CsCO_3_ (20.5 mmol, 2 equiv.), 374 mg Pd(dppf)Cl_2_ (0.512 mmol, 0.05 equiv.), 370 µL H_2_O (20.5 mmol, 2 equiv.) and 3.66 g ((4-bromobenzyl)thio)triisopropylsilane (10.2 mmol, 1 equiv.) and dissolved in degassed 1,4-dioxane (30 mL). Then, the reaction mixture was stirred at 90 °C for 20 h. After complete consumption of the starting material (reaction monitoring via GC-MS), the reaction mixture was cooled to r.t.. The mixture was taken up in H_2_O (30 mL) and extracted with EtOAc (4 x 30 mL). The combined organic phases were dried over Na_2_SO_4_, filtered and concentrated under reduced pressure. The crude product was purified via flash column chromatography (500 mL SiO_2_, 20.0 x 6.0 cm, cyclohexane:EtOAc = 4:1 (v/v) to cyclohexane:EtOAc = 1:1 (v/v)).

**Yield**: 2.99 g (83%), beige, waxy solid, C_21_H_31_NSSi [357.63 g/mol].

**TLC:** R_f_ = 0.45 (cyclohexane:EtOAc = 1:1; UV, KMnO_4_).

**GC-MS**: (EI, 70 eV; MT_50_S): t_R_ = 9.80 min; *m/z* (%): 357 (10), 314 (49), 168 (100), 139 (3), 115 (5).

**^1^H NMR** (300 MHz, CD_2_Cl_2_): δ 8.68 – 8.57 (m, 2H), 7.62 (d, *J* = 8.1 Hz, 2H), 7.55 – 7.43 (m, 4H), 3.81 (s, 2H), 1.40 – 1.25 (m, 3H), 1.16 (d, *J* = 7.2 Hz, 18H) ppm.

**^13^C NMR** (76 MHz, CD_2_Cl_2_): δ 150.8, 148.3, 142.8, 137.2, 129.8, 127.6, 121.9, 30.3, 18.9, 13.3 ppm.

4) **(4-(Pyridin-4-yl)phenyl)methanethiol**

Two batches have been prepared consecutively as described in the following procedure:

In an argon-flushed 500 mL two-necked round-bottom flask, equipped with a Teflon-coated magnetic stirring bar, a mixture of MeOH (150 mL) and 6M aq. HCl (30 mL) were degassed by passing a stream of nitrogen through the solution for 1 h. Afterwards, 2.60 g 4-(4-(((triisopropylsilyl)thio)methyl)phenyl)pyridine (7.27 mmol, 1 equiv.) were added and the mixture was heated under reflux for 20 h. The reaction mixture was allowed to cool to r.t. and diluted with a mixture of MeOH and H_2_O (150 mL; 1:1 (v/v)). The mixture was washed with *n*-pentane (1 x 50 mL, 2 x 100 mL) to remove apolar impurities before the MeOH was removed under reduced pressure. The resulting aqueous solution was again degassed by sonication and passing a stream of nitrogen through the solution for 15 min. Afterwards, the pH of the solution was adjusted to pH ~6 by the addition of solid trisodium citrate dihydrate. The slurry was immediately extracted with DCM (3 x 100 mL). The combined organic phases were dried over Na_2_SO_4_, filtered and concentrated under reduced pressure. The crude product was dissolved in boiling, degassed methyl cyclohexane (250 mL), filtered hot through a Schlenk frit and left for crystallization. The crystals were collected by filtration, washed with *n*-pentane and dried *in vacuo*. Further purification was performed by evaporation and resublimation on a cooling finger (105 °C, 0.15 mbar).

**Yield**: 2.09 g (53% (2 batches)), colourless solid, C_12_H_11_NS [201.29 g/mol].

**GC-MS**: (EI, 70 eV; MT_50_S): t_R_ = 6.97 min; *m/z* (%): 201 (18), 168 (100), 139 (8), 63 (5).

**m.p.** = 62-64 °C.

**^1^H NMR** (400 MHz, CDCl_3_): δ 8.67 – 8.62 (m, 2H), 7.62 – 7.57 (m, 2H), 7.52 – 7.47 (m, 2H), 7.46 – 7.41 (m, 2H), 3.79 (d, *J* = 7.6 Hz, 2H), 1.81 (t, *J* = 7.6 Hz, 1H; -S**H**) ppm.

**^13^C NMR** (101 MHz, CDCl_3_): δ 150.3, 148.0, 142.4, 136.9, 128.9, 127.4, 121.6, 28.7 ppm.

## 2.4. **Characterization techniques**

### 2.4.1. **Grazing Incidence Small Angle X-Ray Scattering (GIWAXS) Experiments**

GIWAXS measurements of the synthesized samples prepared under different stoichiometric conditions were performed at the Austrian SAXS beamline at ELETTRA, Trieste. An X-ray beam with a wavelength of 1.54 Å (beam energy 8 keV) was used with a sample-to-detector distance of 260 mm, providing a *q*-range of 0.01 < *q* < 29.21 nm⁻¹, where *q* denotes the magnitude of the scattering vector. The beam size was 0.1 × 1 mm (v × h). The angular scale of the detector was calibrated using silver behenate. Samples were mounted on a motorized stage with a resolution of 0.001°, and the incident grazing angle was set to 1.6°.

The 2D GIWAXS pattern were acquired in triplicates with an exposure time of 30 s each. A Pilatus3 1M detector (Dectris Ltd., Baden, Switzerland; active area 169 × 179 mm², pixel size 172 µm) was used, and the detector images were processed using SAXSDOG, a software developed at the Austrian SAXS beamline for automatic data reduction.^[29]^ For data analysis, radially integrated diffraction patterns, out-of-plane and in-plane cuts were considered. The cut margin for the out-of-plane and in-plane integration was set to 10 pixels. The integrated data were further processed using IGOR Pro (Wavemetrics, Inc., Lake Oswego, OR). Crystalline phases were identified by comparing the Bragg peaks positions of the respective samples with bulk diffraction patterns of the guest-free single-crystal DMOF structure (see Figure S6).^[30]^

The water vapor stability of the Zn_2_(BDC)_2_(DABCO) thin films was investigated under the same GIWAXS conditions (SSD) using a custom-built humidity chamber.^[31]^ Prior to sample insertion, the chamber was dried and sample alignment was carried out under dry conditions (RH < 5%). Subsequently, the relative humidity was increased to a target value of 90% using controlled humidity ramps of 1%, 2%, and 10%, with the holding time of 1 h once the targeted humidity was reached. Throughout the humidification experiment, in-situ GIWAXS patterns were acquired continuously with an exposure time of 10 s to monitor the structural response of the films in real time.

### 2.4.2. **IR spectromicroscopy**

Infrared measurements were performed at the SISSI-Bio offline end station of the SISSI beamline at Elettra Sincrotrone Trieste. The infrared spectromicroscopy setup is equipped with a grazing-angle objective (GI-IR), which is essential for the analysis of thin films supported on metallic or highly reflective substrates. Spectra were acquired in slow acquisition mode by averaging 128 scans at a spectral resolution of 2 cm^-1^ and a scanner speed of 60 kHz. The aperture were set to 75 × 75 µm^2^ to achieve an optimal signal-to-noise ratio and enable detection of subtle spectral variations. A broadband mercury cadmium telluride detector (MCT-B), operating in the 3800–400 cm^-1^ spectral range, was used for signal detection. All the collected spectra were pre-processed with Bruker software OPUS 9.7. The corresponding background reference spectrum was manually subtracted for minimizing the contribution of water and CO_2_ to the final spectra.

### 2.4.3. **SEM and EDS measurements**

Morphologies of samples were investigated by a scanning electron microscope (FIB–SEM; Aquilos2, Thermo Fisher Scientific, The Netherlands) and cross-sectional analyses were carried out using a focused ion beam in combination with scanning electron microscopy (FIB–SEM; Helios G5 UC, Thermo Fisher Scientific, The Netherlands) equipped with a multi gas injection system (GIS) and an Ultim Max 65 energy-dispersive X-ray spectroscopy (EDS) detector (Oxford Instruments, UK). Prior to analysis, the samples were sputter-coated with a 6 nm platinum (Pt) layer using a PECS 682 system (Gatan, USA) to minimize surface charging.

### 2.4.4. **ToF-SIMS measurements**

Time-of-flight secondary ion mass spectrometry (ToF-SIMS) measurements were performed using a M6 instrument (IONTOF GmbH, Münster, Germany) equipped with a bismuth liquid metal ion gun (LMIG) for analysis and an argon gas cluster ion beam (GCIB) for sputtering. Measurements were conducted in negative ion mode. For spectral acquisition and imaging, a pulsed Bi_3_^+^ primary ion beam was employed as the analysis source. Depth profiling experiments were carried out using an Ar_2000_^+^ GCIB operated at an acceleration energy of 2.5 keV and a sputter current of 1 nA. The sputter beam was rastered over an area of 500 µm by 500 µm, while secondary ions were collected only from the central analysis region over an area of 300 µm by 300 µm (128 by 128 pixels) to avoid contributions from crater edges. Analysis and sputtering were alternated in a cyclic manner to obtain depth-resolved chemical information. High lateral resolution two-dimensional chemical imaging was performed using the delayed extraction analyzer mode in combination with the fast-imaging LMIG mode. Imaging was conducted over a field of view of 52 µm by 52 µm (512 by 512 pixels). Charge compensation was applied during all measurements using a low-energy electron flood gun (20 eV). Mass calibration was performed using the signals for C_3_^–^ at mass-to-charge (*m*/*z*) 36.00, C_4_^–^ at *m*/*z* 48.00, and C_5_^–^ at *m*/*z* 60.00. All data acquisition, visualization, and post-processing were carried out using SurfaceLab 7.6 software (IONTOF GmbH).

Two-dimensional ToF-SIMS images were acquired using the delayed extraction mode combined with fast imaging LMIG mode. This approach provides high mass resolution (~10,000) while maintaining lateral resolution of ~100 nm. The coverage of ZnOH^-^ was calculated as the fraction of pixels with intensities above a threshold value of 1 to the total number of pixels.

### 2.4.5. **3D Profilometry**

The surface topography and the analysis of the sputter crater depths formed during GCIB sputtering in the ToF-SIMS measurements were evaluated using a DektakXT stylus profilometer controlled with Visio64 software program (Bruker, Karlsruhe, Germany). The resulting 3D surface profiles were subsequently processed and evaluated with MountainsMap^®^ Imaging Topography software (Digital Surf, Besançon, France). During post-processing, levelling was applied to correct for sample tilt and align the measured surface to a common reference plane, while form removal was used to subtract the large-scale surface shape or curvature, enabling clearer evaluation of the crater geometry and local topographic features. Three-dimensional surface topography maps were acquired over areas of 3 by 3 mm^2^ and 1.5 by 2.5 mm^2^.

# 3. **LPE Spin-Coating Workflow**

**Figure S1.** **Process overview of the automatic deposition step.** (a) Photograph of the spin-assisted LPE-LbL setup; (b) The tube holder moves according to the programmed sequence to precisely dispense the solution at the center of the spinned wafer. (c) Outline of the programmed sequence of steps representing one deposition cycle.

**Figure S2**. **Quality control during the fabrication process.** (a) Contact angle measurements show a progressive loss of order in the SAM layer within time. Each data point represents the average of three independent samples, and the error bands indicate the presence of structural variability even under optimal functionalization conditions.^[32]^ However, functionalized substrates used within the first 48 h enable reproducible growth of highly oriented, homogeneous MOF films. (b) UV-Vis absorption spectra of a 0.1 mM BDC solution and the working solution after addition of DABCO at a 1:3 ratio (left). The band shift at 284 nm limits accurate concentration determination in the mixed-ligand system; therefore, only BDC was quantified using its calibration curve at this wavelength (right). (c) GIWAXS, out-of-plane cuts, illustrating the loss of crystallinity when the sample is allowed to dry during spinning as indicated by the weakening of the (001) reflection. (d) The GI-IR spectra contain characteristic asymmetric (1646 cm^-1^) and symmetric (1394 cm^-1^) stretching vibrations of coordinated carboxylic groups indicating MOF formation and confirm that the chemical composition of the samples remains unchanged under different drying conditions.

# 4. **Assessment of film uniformity and fabrication reproducibility**

**Figure S3.** **IR and GIWAXS uniformity analysis.** (a) 3D projection of the intensity of the asymmetric stretching vibrations of the carboxylate group in the range of 1667-1630cm^-1^ over the full sample area equal to 1 cm x 1 cm (BDC:DABCO= 1:3) with 60 cycles, indicating formation of MOF. (b) IR-data for the samples with BDC:DABCO=1:2, 1:4, 1:5 molar ratios with 60/120. To probe the homogeneity, each wafer was first imaged and subsequently probed at 3-4 different locations (center and edges) to compare spectral features across distinct surface regions. Colour of the spectra in the central column corresponds to the colour of the point in the image. Samples prepared with 60 deposition cycles and varying reagent molar ratios exhibit high uniformity across the entire sample, including also the edges. In contrast, increasing the number of cycles to 120 results in a slight loss of homogeneity and the appearance of by-products at the wafer edges. (c) GIWAXS measurements across the sample, where 0 mm is the position at the centre of the sample. Integrated out-of-plane intensity versus horizontal positions scanning the entire sample confirm higher uniformity of the 60 cycles samples.

**Figure S4.** IR spectra of samples prepared with varying BDC:DABCO molar ratios and deposition cycles confirm the reproducibility of the LbL-LPE spin-coating method. The band at 1294 cm⁻^1^, assigned to unreacted DABCO, appears only in the 1:5 samples and in the 1:4 samples after 120 cycles due to increased material deposition. In contrast, the band at 1479 cm⁻^1^ corresponds to coordinated DABCO and is weak in samples synthesized with a 1:2 ligand ratio, 60 cycles.

# 5. **DFT calculations**

To reproduce the experimental spectrum as accurately as possible and enable reliable assignment of the bands to their vibrational modes, DFT calculations were performed.

## 5.1. **Computational Details**

The initial model structures of Zn_2_BDC_2_DABCO were generated based on the computational structures of Griffith et al.^[33]^ using VESTA and the GUI of the atomic simulation environment (ASE) to yield a $\sqrt{2}\times\sqrt{2}\times1$ supercell.^[34]^

DFT calculations were performed with FHI-aims^[35–40]^ (version 210716_2) employing light settings^[35]^ and the PBE functional^[41]^ with the many-body dispersion correction^[42,43]^ (PBE+MBD/light). Relativistic effects were treated by the scalar atomic ZORA approximation as implemented in FHI-aims. The k-grids for both the periodic DFT calculations and the MBD energies and forces were set to always satisfy $nq>30 Å$, with $q$ being the cell length in the respective direction and $n$ being the number of k-points in that direction. Convergence criteria were set to 10^-6^ eV for the energy, 10^-4^ eV/Å for the gradient, 10^-3^ for the sum of eigenvalues and 10^-5^ for the density.

Optimizations with fixed cell parameters were performed with the FHI-AIMS implementation of the BFGS optimizer with an energy tolerance of 0.001 eV and enforcing a maximum force of 0.005 eV/Å (Figure SI1 to SI3).

Full cell optimizations were performed by employing the BFGS implementation and ExpCellFilter class of ASE,^[35]^ enforcing a maximum force of 0.005 eV/Å.

Vibrational spectra were calculated by the finite displacement approach as implemented in Phonopy.^[44,45]^ The respective calculations were performed on the same $\sqrt{2}\times\sqrt{2}\times1$ supercells with finite displacements of 0.005 Å. The optimized geometries were checked to not exhibit any imaginary modes at the Γ-point. Born effective charges were calculated *via* a finite differences approach using the BEC.py script of FHI-aims (displacements were chosen to be 0 and 0.005 Å and the polarization k-grid along the reciprocal lattice vectors was set to *k_x_* = 8 x 2 x 2, *k_y_* = 2 x 8 x 2 and *k_z_* = 2 x 2 x 8). The dielectric tensors were calculated with Density-functional perturbation theory (DFPT), retaining all other relevant settings. Infrared spectra at the individual volumes were obtained *via* the Phonopy-Spectroscopy code.^[46,47]^

Animation files for the Phonon modes were generated with Phonopy and visualized with Jmol.^[44,45,48]^ Similarly, the geometries depicted below were rendered in Jmol.

## 5.2. **Detailed Information regarding Computations**

Firstly, full cell optimizations of Zn_2_BDC_2_DABCO were performed for five different model structures (**I** to **V**). The resulting geometries (Figures S5 to S7) exhibit only subtle structural differences, especially regarding DABCO orientation and the arrangement of the coordination sphere metal nodes (the resulting cell parameters are summarized in Table S2). The obtained volumes are in good agreement with the experimental X-ray structure (with deviations around 0.6%, see Table S1) and the electronic energies of the structures differ only marginally and are within the error of the method (the maximum absolute deviation is 2.28$\times$10^-3^ eV or 0.22 kJ/mol). Gratifyingly, the obtained IR-spectra display almost no dependence on the structural model used, as they are visually virtually indistinguishable (Figures S10).

Secondly, an alternative way to obtain calculated IR spectra that accurately reflect the experimental data and, thus, allow reliable assignment of the individual bands, is to fix the cell parameters to their experimentally determined values. Hence, in addition to the above outlined full cell optimization approach we also opted to pursue this strategy to assess the robustness of our calculated IR spectra and their dependence on the chosen computational model. Towards this, all model structures **I** to **V** were also optimized with the cell parameters fixed to the experimentally determined values (termed **I-exp.** to **V-exp.**).^[33]^ Of the five structures, only **I-exp.** and **V-exp.** did not exhibit an imaginary frequency at the Γ-point. Their geometries are displayed and compared to their respective parent structures **I** and **V** in Figures S8 and S9. Importantly, the IR-spectra obtained for **I-exp.** and **V-exp.** only slightly differ from the ones obtained from the fully optimized model structures **I** to **V** (Figures S5- S7), rendering the qualitative conclusions obtained independent of the chosen computational approach, *i.e.*, full cell optimization versus internal cell optimization with cell parameters fixed to their experimental values. This also applies to the peak assignment, see section 5, Table S3. For the main text of the publication, the IR-spectrum obtained from model structure **V-exp.** was chosen.

**Table S2**. Cell volumes and electronic energies of the calculated model structures and comparison to the experiment (entry “Exp.”).

| structure | V [Å^3^] | E [eV] | ΔE [eV] | ΔE vs. **I** [kJ/mol] | ΔV vs exp. [Å^3^] | ΔV vs exp. [%] |
| --- | --- | --- | --- | --- | --- | --- |
| **I** | 2308.236 | -281489.208635371 | 0.00 | 0.00 | 13.438 | 0.59 |
| **II** | 2309.324 | -281489.208479918 | 1.04$\times$10^-4^ | 0.01 | 14.526 | 0.63 |
| **III** | 2310.473 | -281489.207031581 | 1.55$\times$10^-3^ | 0.15 | 15.676 | 0.68 |
| **IV** | 2310.421 | -281489.206387599 | 2.28$\times$10^-3^ | 0.22 | 15.624 | 0.68 |
| **V** | 2310.590 | -281489.207266224 | 1.35$\times$10^-3^ | 0.13 | 15.792 | 0.69 |
| **I-exp.** | 2294.797 | -281489.149519445 | 5.91$\times$10^-2^ | 5.70 | 0.000 | 0.00 |
| **V-exp.** | 2294.797 | -281489.150958683 | 5.77$\times$10^-2^ | 5.57 | 0.000 | 0.00 |
| Exp. | 2294.797 | n.a. | n.a. | n.a. | 0.000 | 0.00 |

**Table S3.** Cell parameters of the calculated model structures and comparison to the experiment (entry “Exp.”).

| structure | a [Å] | b [Å] | c [Å] | α [°] | β [°] | γ [°] |
| --- | --- | --- | --- | --- | --- | --- |
| **I** | 15.724 | 15.419 | 9.523 | 91.057 | 89.123 | 90.010 |
| **II** | 15.610 | 15.534 | 9.524 | 90.490 | 89.358 | 89.999 |
| **III** | 15.581 | 15.565 | 9.527 | 89.714 | 89.896 | 89.997 |
| **IV** | 15.634 | 15.514 | 9.526 | 90.203 | 89.781 | 90.006 |
| **V** | 15.595 | 15.551 | 9.527 | 89.761 | 89.853 | 89.998 |
| **I-exp.** | 15.455 | 15.455 | 9.608 | 90.000 | 90.000 | 90.000 |
| **V-exp.** | 15.455 | 15.455 | 9.608 | 90.000 | 90.000 | 90.000 |
| Exp. | 15.455 | 15.455 | 9.608 | 90.000 | 90.000 | 90.000 |

|  |  |
| --- | --- |
| **I** | **II** |
|  |  |
| **III** | **IV** |
|  |  |
| **V** |  |

**Figure S5.** Rendered images of the 3D structures of models **I** to **V** (view along the c-axis).

|  |  |
| --- | --- |
| **I** | **II** |
|  |  |
| **III** | **IV** |
|  |  |
| **V** |  |

**Figure S6.** Rendered images of the 3D structures of models **I** to **V** (view along the b-axis).

|  |  |
| --- | --- |
| **I** | **II** |
|  |  |
| **III** | **IV** |
|  |  |
| **V** |  |

**Figure S7.** Rendered images of the 3D structures of models **I** to **V** (view along the a-axis).

|  |  |
| --- | --- |
| **I** | **I-exp.** |
|  |  |
| **I** | **I-exp.** |
|  |  |
| **I** | **I-exp.** |

**Figure S8.** Comparison of the 3D structures of **I** (left) and **I-exp.** (right).

|  |  |
| --- | --- |
| **V** | **V-exp.** |
|  |  |
| **V** | **V-exp.** |
|  |  |
| **V** | **V-exp.** |

**Figure S9.** Comparison of the 3D structures of **V** (left) and **V-exp.** (right).

**Figure S10.** Overlay of the calculated IR-spectra for the investigated model structure.

# 6. **Calculated vs. experimental IR spectra and vibrational peaks assignment**

To compare the two computational model approaches and obtain a conclusion that is qualitatively independent of the chosen model, the assignment of the peaks was done on structures **IV** and **V-exp.,** representative of the two above outlined computational model approaches, i.e., full cell optimization versus internal cell optimization with cell parameters fixed to their experimental values. The computational spectra of **IV** and **V-exp.** both well reflect the experimental spectrum (Figure S11). The individual bands in the experimental spectrum were assigned to the respective type of mode by selecting the most intense individual vibrations making up the corresponding bands in the theoretical spectra of **IV** and **V-exp.**, subsequent visual inspection in the animation file and, where possible, compared to literature.^[49–51]^ Gratifyingly, the assignment of the peaks does not differ between **IV** and **V-exp.**, rendering the obtained picture independent of the computational model approach.

**Figure S11.** Comparison of calculated structures **IV** and **V-exp.** with the experimental spectra of the LbL-LPE film (optimal conditions) and the bulk powder. The calculated spectrum matches the bulk powder spectrum, while the oriented structure has a different intensity distribution between asymmetric and symmetric carboxylic group vibrations. This is known to occur for oriented structures, as shown for SSH-fabricated Cu_2_BDC_2_DABCO films by Baumgartner *et al*.^[52]^ The calculated IR spectrum was scaled (≈0.9) along the frequency axis to correct for the systematic overestimation of vibrational frequencies inherent to the harmonic approximation used in theoretical calculations, enabling better agreement with the experimental spectrum.^[53]^

**Table S4.** Assignment of the vibrational bands in the calculated and experimental spectra.

| **Frequency in experimental spectrum, cm^-1^** | **Frequency in calculated spectrum IV, cm^-1^** | **Frequency in calculated spectrum V-exp., cm^-1^** | **Vibrations** |
| --- | --- | --- | --- |
| 1646 | 1610 | 1615 | COO^-^ stretching asymmetric |
| 1588 | 1555 | 1559 | BDC  stretching asymmetric |
| 1479 | 1478 | 1480 | DABCO  CH_2_ bending |
| 1391 | 1358 | 1363 | BDC  stretching symmetric |
|  |  |  | COO^-^ stretching symmetric |
|  |  |  | DABCO  NC_3_ stretching symmetric  CH_2_ bending |
| 1092 | 1083 | 1085 | BDC  С-H scissoring |
| 1021 | 1038 | 1037 | DABCO  C-C stretching asymmetric |
| 1022 | 995 | 992 | DABCO  NC_3_ scissoring |
| 812 | 810 | 810 | DABCO  C-C stretching symmetric |
| 744 | 721 | 718 | BDC  C-H wagging |

# 7. **GIWAXS Characterization of Zn₂(BDC)₂(DABCO) Films**

**Figure S12. Crystalline phase identification and homogeneity of the films.** (a) The LbL-LPE films show preferential orientation in the out-of-plane and in-plane direction denoted by the black arrows. The cuts employed for data analysis for the out-of-plane (cut margin 10 pixels,) and in-plane (cut margin 10 pixels) over the entire *q*-range (*q* < 29.21 nm⁻¹) are indicated by the black dotted line. (b) Comparison with the powder diffraction pattern of the sample prepared under optimal conditions confirms that the dominant reflection corresponds to the (001)-oriented MOF phase. The presence of the (110), (111), and (101) reflections is related to the existence of tilted crystallites. (c) Out-of-plane integration of the GIWAXS intensity of MOF thin films of 60 synthesis cycles with different molar ratios on the MHDA (left) and PP1 (right) functionalized SAM layers. These data confirm that for the molar ratio BDC:DABCO = 1:3 most of the crystallites are oriented along (001). (d) Out-of-plane integration of the GIWAXS patterns for samples with 120 fabrication cycles shows the (001) reflection, indicating that increasing the number of cycles up to 120 suppresses the crystalline orientation. (e) In-plane integration of the GIWAXS patterns of samples with the optimum ratio Zn-BDC-DABCO=1:1:3 of different synthesis cycles on MHDA layer, shows absence of a (001) in-plane alignment, which confirms that that the film is textured rather than epitaxially ordered.

# 8. **Surface and cross-section morphology**

**Figure S13. SEM and EDS Analysis of MOF Film Morphology.** (a) SEM images of FIB-cut samples of 60 and 120 synthesis cycles with the molar ratio Zn:BDC:DABCO = 1:1:3 on MHDA self-assembled monolayer. (b) EDS spectra collected from two regions of the film surface in (a): on a crystal (inset, Spectrum 2) and in the area between crystals (inset, Spectrum 3). The EDS data indicate that the regions between the crystals contain no metal, suggesting that MOF growth follows an island-like mechanism. (c) SEM images of the amorphous samples with the molar ratio Zn:BDC:DABCO=1:1:1.

# 9. **Thin film thickness measurement with 3D profilometry**

The sputter crater depth was determined by 3D stylus profilometry from the height difference between the sputtered region and the surrounding intact surface. For each crater, the central area away from the edges was selected as the sputtered plane. These regions are marked as X1 and X2 for the sample with BDC:DABCO = 1:3 and as X3 and X4 for the sample with BDC:DABCO = 1:5. The non-sputtered surface was defined using rectangular reference areas (Y) on the surrounding intact surface. The average height of the Y regions established the reference plane, while the corresponding X region represented the crater floor. The resulting crater depths correspond to the material thickness removed during GCIB sputtering under the applied ToF-SIMS depth-profiling conditions.

**Figure S14. Sample Thickness Determination.** The thickness of samples with 1:3 and 1:5 BDC:DABCO ligand ratios on the MHDA layer was determined by creating two craters on each sample using ToF-SIMS with GCIB sputtering, followed by measuring the height differences across the surface using 3D profilometry. Three-dimensional surface topography maps (a), (c) indicate the crater positions, while the corresponding selected surface height profiles (b), (d) are shown on the right and were used to determine the averaged crater depth extracted from the topographic data.

# 10. **Degree of orientation and Hermans orientation parameter calculations**

The degree of orientation (DO) and the Hermans orientation parameter (HOP) were determined by analyzing the azimuthal intensity distribution of the sharp, well-defined (001) diffraction spot, following established analysis protocols.^[16,54–56]^ For a simple estimation, the flat component of the azimuthally integrated intensity was attributed to an isotropic distribution of crystallites, while the remaining intensity was assigned to preferentially oriented crystallites. This ordering metric does not account for the angular width of the preferential orientations, which can be evaluated from the azimuthal full width at half maximum (FWHM) of the respective peak.

Experimental data were reshaped into 2D diffractograms in reciprocal space using the open-access software GIXSGUI (Figure S15a).^[57]^ To remove non-sample-related scattering, background contributions from air, the gold substrate, and other sources were estimated by taking azimuthal cuts above and below the peak, averaging them, and subtracting the result from the central cut. For quantitative texture analysis, two corrections were applied to the pole figure data (Figure S15b). First, the signal near 90° (the horizon) was excluded, as it was identified as the Yoneda peak.^[58]^ Second, the missing wedges at χ = 0° and 37–54° were accounted for, by fitting using a Gaussian and linear functions, and the full angular range from 0° to 90° was analyzed (Figure S15c).

Correction of the pole figure was performed by multiplying the measured intensity by sin(χ). This correction converts the measured scattering intensity around *q_z_* into the total scattering signal that would be expected if all crystallites in the 2D and 3D powder textures were accessible at a given χ. Subsequent normalization of the intensity per area under the diffraction peak enables direct comparison between measurements independent of geometric factors.

The degree of orientation is calculated by

$$DO \left( \% \right)=\frac{A_{total}-A_{isotropic}}{A_{total}}\cdot100$$

where $A_{total}$ is the area below the integrated intensity as a function of azimuthal angle 𝜒, and $A_{isotropic}$ is the isotropic fraction as shown in Figure S15d.^[54,55]^

Hermans orientation parameter (HOP) is calculated by the formulas

$$\left\langle{cos}^{2}\chi\right\rangle= \frac{\sum I(\chi){cos}^{2}\chi}{\sum I(\chi)}$$

$$HOP=\frac{3\left\langle{cos}^{2}\chi\right\rangle-1}{2}$$

where $I(\chi)$ is intensity at azimuthal angle 𝜒.^[59]^

HOP ranges from -0.5 (perfectly perpendicular alignment) to 1 (perfectly aligned along the reference axis) and 0 is fully isotropic distribution.

Although obtaining a complete pole figure down to γ = 0° is not possible for reflections along *q_z_* and corrections were therefore applied, the presented methodology provides a few-step approach for GIWAXS data analysis that enables reliable comparison of different datasets and assessment of synthesis reproducibility.

**Figure S15. GIWAXS data processing.** (a) Reshaped GIWAXS image showing the intense (001) reflection and corresponding azimuthal cuts of the background above and below the reflection, as well as the main diffraction peak. (b) Background intensity subtraction. (c) Corrections applied to the pole figure data, including missing wedge accounting and geometric weighting. (d) Corrected and area-normalized pole figure intensities highlighting the isotropic crystallite contribution.

**Figure S16.** Degree of orientation (DO) versus full width at half maximum (FWHM) for LbL-LPE films grown on MHDA (a) and PP1 (b). The plot illustrates the highest degree of orientation (DO > 80%) for samples with BDC:DABCO molar ratios of 1:2 and 1:3 after 60 deposition cycles, consistent with the observed SEM data (see Figure 2 in the main manuscript).

**Table S5.** Overview of the mean values of the DO, HOP and FWHM for samples with different linker-to-linker ratio.

| **Ratio** | **SAM** | **Cycles** | **DO** | **HOP** | **FWHM** | **No. of samples** |
| --- | --- | --- | --- | --- | --- | --- |
| **1:2** | **MHDA** | 60 cycles | 68.18 ± 21.19% | 0.86 ± 0.14 | 4.49 ± 1.08 | 3 |
|  |  | 120 cycles | 81.69 ± 5.94% | 0.85 ± 0.03 | 5.23 ± 1.19 | 2 |
|  | **PP1** | 60 cycles | 61.58 ± 18.42% | 0.94 ± 0.02 | 4.04 ± 0.54 | 3 |
|  |  | 120 cycles | 90.27 ± 0.28% | 0.86 ± 0.01 | 11.36 ± 0.87 | 2 |
| **1:3** | **MHDA** | 60 cycles | 88.64 ± 7.94% | 0.93 ± 0.04 | 5.18 ± 0.98 | 6 |
|  |  | 120 cycles | 89.64 ± 2.64% | 0.83 ± 0.03 | 17.42 ± 1.93 | 3 |
|  | **PP1** | 60 cycles | 61.30 ± 23.70% | 0.95 ± 0.03 | 4.05 ± 0.27 | 5 |
|  |  | 120 cycles | 90.77 ± 5.59% | 0.88 ± 0.03 | 9.39 ± 2.75 | 3 |
| **1:4** | **MHDA** | 60 cycles | 83.33 ± 9.23% | 0.87 ± 0.07 | 7.20 ± 1.83 | 3 |
|  |  | 120 cycles | 87.51 ± 3.50% | 0.78 ± 0.01 | 10.61 ± 1.31 | 2 |
|  | **PP1** | 60 cycles | 88.69 ± 16.91% | 0.82 ± 0.13 | 11.02 ± 2.08 | 3 |
|  |  | 120 cycles | 92.63 ± 3.06% | 0.84 ± 0.01 | 9.62 ± 0.28 | 2 |
| **1:5** | **MHDA** | 60 cycles | 83.77 ± 0.20% | 0.81 ± 0.01 | 10.79 ± 2.36 | 2 |
|  |  | 120 cycles | 87.91 ± 0.07% | 0.80 ± 0.01 | 10.24 ± 0.92 | 2 |
|  | **PP1** | 60 cycles | 78.19 ± 2.79% | 0.79 ± 0.01 | 8.18 ± 1.67 | 2 |
|  |  | 120 cycles | 70.40 ± 32.84% | 0.81 ± 0.02 | 9.62 ± 0.28 | 2 |

# 11. **Hydrolytic Stability of Zn_2_(BDC)_2_(DABCO) Thin Films**

**Figure S17**. **(a)** GI-IR spectra of Zn-BDC confirms that the band appearing at 1589 cm^-1^ in the humidified films corresponds to the formation of Zn-BDC. This reference sample was intentionally prepared using the same spin-assisted LbL-LPE fabrication approach as the Zn_2_(BDC)_2_(DABCO) films, while omitting the DABCO linker to selectively form the Zn-BDC phase. **(b)** *Ex-situ* GIWAXS characterization of highly oriented Zn_2_(BDC)_2_(DABCO) thin films (Zn:BDC:DABCO = 1:1:3) exposed to 80% relative humidity after 24 and 48 h of humidity.

**Figure S18**. *In-situ* characterization of highly oriented Zn_2_(BDC)_2_(DABCO) thin films (Zn:BDC:DABCO = 1:1:3) during a controlled humidification protocol to 90% relative humidity, using humidity ramps of 1, 2, and 10 %/min, with an equilibration time of 1 h at final humidity. (a) Evolution of the (001) reflection in the GIWAXS patterns, demonstrating a reversible shift toward lower *q* values during humidification for all humidity ramps. (b) GI-IR spectra recorded after completion of the humidification experiment, confirming that the chemical structure of Zn_2_(BDC)_2_(DABCO) remains unchanged. (c) SEM images of the films after the humidity experiments, showing only minor surface degradation for the sample subjected to the slowest humidity ramp (1%/min), without detectable changes in the overall crystallinity or chemical composition. (d) Full GIWAXS patterns recorded before and after the humidification experiment.

### **References**

(1)	Genesio, G.; Maynadié, J.; Carboni, M.; Meyer, D. Recent Status on MOF Thin Films on Transparent Conductive Oxides Substrates (ITO or FTO). *New J. Chem.* **2018**, *42* (4), 2351–2363. https://doi.org/10.1039/C7NJ03171H.

(2)	Bétard, A.; Fischer, R. A. Metal–Organic Framework Thin Films: From Fundamentals to Applications. *Chem. Rev.* **2012**, *112* (2), 1055–1083. https://doi.org/10.1021/cr200167v.

(3)	Sabzehmeidani, M. M.; Gafari, S.; jamali, S.; Kazemzad, M. Concepts, Fabrication and Applications of MOF Thin Films in Optoelectronics: A Review. *Applied Materials Today* **2024**, *38*, 102153. https://doi.org/10.1016/j.apmt.2024.102153.

(4)	Zhang, Y.; Chang, C.-H. Metal–Organic Framework Thin Films: Fabrication, Modification, and Patterning. *Processes* **2020**, *8* (3), 377. https://doi.org/10.3390/pr8030377.

(5)	Liu, Y.; Zhao, Z.; Li, M.; Zhao, Z. Metal–Organic Framework Thin Films: Review of Their Room-Temperature Synthesis and Applications. *J. Mater. Chem. C* **2024**, *12* (10), 3394–3409. https://doi.org/10.1039/D3TC04366E.

(6)	Choe, M.; Lee, H.; Choi, H. C. Vapor‐phase Synthesis of MOF Films. *Bulletin Korean Chem Soc* **2024**, *45* (7), 584–592. https://doi.org/10.1002/bkcs.12854.

(7)	Scheurle, P. I.; Mähringer, A.; Biewald, A.; Hartschuh, A.; Bein, T.; Medina, D. D. MOF-74(M) Films Obtained through Vapor-Assisted Conversion—Impact on Crystal Orientation and Optical Properties. *Chem. Mater.* **2021**, *33* (15), 5896–5904. https://doi.org/10.1021/acs.chemmater.1c00743.

(8)	Claire, F. J.; Solomos, M. A.; Kim, J.; Wang, G.; Siegler, M. A.; Crommie, M. F.; Kempa, T. J. Structural and Electronic Switching of a Single Crystal 2D Metal-Organic Framework Prepared by Chemical Vapor Deposition. *Nat Commun* **2020**, *11* (1), 5524. https://doi.org/10.1038/s41467-020-19220-y.

(9)	Rodríguez-Hermida, S.; Kravchenko, D. E.; Wauteraerts, N.; Ameloot, R. Vapor-Assisted Powder Synthesis and Oriented MOF-CVD Thin Films of the Metal–Organic Framework HKUST-1. *Inorg. Chem.* **2022**, *61* (45), 17927–17931. https://doi.org/10.1021/acs.inorgchem.2c02490.

(10)	M. V., V.; Nageswaran, G. Review—Direct Electrochemical Synthesis of Metal Organic Frameworks. *J. Electrochem. Soc.* **2020**, *167* (15), 155527. https://doi.org/10.1149/1945-7111/abc6c6.

(11)	Mueller, U.; Schubert, M.; Teich, F.; Puetter, H.; Schierle-Arndt, K.; Pastré, J. Metal–Organic Frameworks—Prospective Industrial Applications. *J. Mater. Chem.* **2006**, *16* (7), 626–636. https://doi.org/10.1039/B511962F.

(12)	Cui, X.-Y.; Gu, Z.-Y.; Jiang, D.-Q.; Li, Y.; Wang, H.-F.; Yan, X.-P. In Situ Hydrothermal Growth of Metal−Organic Framework 199 Films on Stainless Steel Fibers for Solid-Phase Microextraction of Gaseous Benzene Homologues. *Anal. Chem.* **2009**, *81* (23), 9771–9777. https://doi.org/10.1021/ac901663x.

(13)	Crivello, C.; Sevim, S.; Graniel, O.; Franco, C.; Pané, S.; Puigmartí-Luis, J.; Muñoz-Rojas, D. Advanced Technologies for the Fabrication of MOF Thin Films. *Mater. Horiz.* **2021**, *8* (1), 168–178. https://doi.org/10.1039/D0MH00898B.

(14)	Falcaro, P.; Okada, K.; Hara, T.; Ikigaki, K.; Tokudome, Y.; Thornton, A. W.; Hill, A. J.; Williams, T.; Doonan, C.; Takahashi, M. Centimetre-Scale Micropore Alignment in Oriented Polycrystalline Metal–Organic Framework Films via Heteroepitaxial Growth. *Nature Mater* **2017**, *16* (3), 342–348. https://doi.org/10.1038/nmat4815.

(15)	Shekhah, O.; Hirai, K.; Wang, H.; Uehara, H.; Kondo, M.; Diring, S.; Zacher, D.; Fischer, R. A.; Sakata, O.; Kitagawa, S.; Furukawa, S.; Wöll, C. MOF-on-MOF Heteroepitaxy: Perfectly Oriented [Zn2(Ndc)2(Dabco)]n Grown on [Cu2(Ndc)2(Dabco)]n Thin Films. *Dalton Trans.* **2011**, *40* (18), 4954. https://doi.org/10.1039/c0dt01818j.

(16)	Klokic, S.; Naumenko, D.; Marmiroli, B.; Carraro, F.; Linares-Moreau, M.; Zilio, S. D.; Birarda, G.; Kargl, R.; Falcaro, P.; Amenitsch, H. Unraveling the Timescale of the Structural Photo-Response within Oriented Metal–Organic Framework Films. *Chem. Sci.* **2022**, *13* (40), 11869–11877. https://doi.org/10.1039/D2SC02405E.

(17)	Sun, Y.; Zhang, R.; Zhao, C.; Wang, N.; Xie, Y.; Li, J.-R. Self-Modified Fabrication of Inner Skin ZIF-8 Tubular Membranes by a Counter Diffusion Assisted Secondary Growth Method. *RSC Adv.* **2014**, *4* (62), 33007–33012. https://doi.org/10.1039/C4RA05182C.

(18)	Kim, J.-O.; Koo, W.-T.; Kim, H.; Park, C.; Lee, T.; Hutomo, C. A.; Choi, S. Q.; Kim, D. S.; Kim, I.-D.; Park, S. Large-Area Synthesis of Nanoscopic Catalyst-Decorated Conductive MOF Film Using Microfluidic-Based Solution Shearing. *Nat Commun* **2021**, *12* (1), 4294. https://doi.org/10.1038/s41467-021-24571-1.

(19)	Sevim, S.; Franco, C.; Liu, H.; Roussel, H.; Rapenne, L.; Rubio‐Zuazo, J.; Chen, X.; Pané, S.; Muñoz‐Rojas, D.; deMello, A. J.; Puigmartí‐Luis, J. In‐Flow MOF Lithography. *Adv Materials Technologies* **2019**, *4* (6), 1800666. https://doi.org/10.1002/admt.201800666.

(20)	Lee, T.; Kim, J.; Park, C.; Kim, H.; Kim, M.; Park, H.; Kim, I.; Ko, J.; Pak, K.; Choi, S. Q.; Kim, I.; Park, S. Large‐Area Synthesis of Ultrathin, Flexible, and Transparent Conductive Metal–Organic Framework Thin Films via a Microfluidic‐Based Solution Shearing Process. *Advanced Materials* **2022**, *34* (12), 2107696. https://doi.org/10.1002/adma.202107696.

(21)	Xiao, Y.-H.; Tian, Y.-B.; Gu, Z.-G.; Zhang, J. Surface-Coordinated Metal-Organic Framework Thin Films (SURMOFs): From Fabrication to Energy Applications. *EnergyChem* **2021**, *3* (6), 100065. https://doi.org/10.1016/j.enchem.2021.100065.

(22)	Shekhah, O.; Wang, H.; Kowarik, S.; Schreiber, F.; Paulus, M.; Tolan, M.; Sternemann, C.; Evers, F.; Zacher, D.; Fischer, R. A.; Wöll, C. Step-by-Step Route for the Synthesis of Metal−Organic Frameworks. *J. Am. Chem. Soc.* **2007**, *129* (49), 15118–15119. https://doi.org/10.1021/ja076210u.

(23)	Shekhah, O.; Wang, H.; Zacher, D.; Fischer, R. A.; Wöll, C. Growth Mechanism of Metal–Organic Frameworks: Insights into the Nucleation by Employing a Step‐by‐Step Route. *Angew Chem Int Ed* **2009**, *48* (27), 5038–5041. https://doi.org/10.1002/anie.200900378.

(24)	Semrau, A. L.; Fischer, R. A. High‐Quality Thin Films of UiO‐66‐NH_2_ by Coordination Modulated Layer‐by‐Layer Liquid Phase Epitaxy. *Chemistry A European J* **2021**, *27* (33), 8509–8516. https://doi.org/10.1002/chem.202005416.

(25)	Zheng, R.; Fu, Z.; Deng, W.; Wen, Y.; Wu, A.; Ye, X.; Xu, G. The Growth Mechanism of a Conductive MOF Thin Film in Spray‐based Layer‐by‐layer Liquid Phase Epitaxy. *Angew Chem Int Ed* **2022**, *61* (43), e202212797. https://doi.org/10.1002/anie.202212797.

(26)	Silva, A. G. V.; Ribeiro, J. O. N.; Vasconcelos, D. C. L.; Weidler, P. G.; Vasconcelos, W. L. Room Temperature and Ambient Pressure Deposition of Cu-BTC MOF on SBA-15 Functionalized Silica Supports by Simple Spray Layer-by-Layer Method. *Materials Today Communications* **2021**, *27*, 102388. https://doi.org/10.1016/j.mtcomm.2021.102388.

(27)	Chernikova, V.; Shekhah, O.; Eddaoudi, M. Advanced Fabrication Method for the Preparation of MOF Thin Films: Liquid-Phase Epitaxy Approach Meets Spin Coating Method. *ACS Appl. Mater. Interfaces* **2016**, *8* (31), 20459–20464. https://doi.org/10.1021/acsami.6b04701.

(28)	Wannapaiboon, S.; Schneemann, A.; Hante, I.; Tu, M.; Epp, K.; Semrau, A. L.; Sternemann, C.; Paulus, M.; Baxter, S. J.; Kieslich, G.; Fischer, R. A. Control of Structural Flexibility of Layered-Pillared Metal-Organic Frameworks Anchored at Surfaces. *Nat Commun* **2019**, *10* (1), 346. https://doi.org/10.1038/s41467-018-08285-5.

(29)	Burian, M.; Meisenbichler, C.; Naumenko, D.; Amenitsch, H. *SAXSDOG* : Open Software for Real-Time Azimuthal Integration of 2D Scattering Images. *J Appl Crystallogr* **2022**, *55* (3), 677–685. https://doi.org/10.1107/S1600576722003685.

(30)	Dybtsev, D. N.; Chun, H.; Kim, K. Rigid and Flexible: A Highly Porous Metal–Organic Framework with Unusual Guest‐Dependent Dynamic Behavior. *Angew Chem Int Ed* **2004**, *43* (38), 5033–5036. https://doi.org/10.1002/anie.200460712.

(31)	Marmiroli, B.; Sartori, B.; Kyvik, A. R.; Ratera, I.; Amenitsch, H. Structural Study of the Hydration of Lipid Membranes Upon Interaction With Mesoporous Supports Prepared by Standard Methods and/or X‐Ray Irradiation. *Front. Mater.* **2021**, *8*, 686353. https://doi.org/10.3389/fmats.2021.686353.

(32)	Singh, M.; Kaur, N.; Comini, E. The Role of Self-Assembled Monolayers in Electronic Devices. *J. Mater. Chem. C* **2020**, *8* (12), 3938–3955. https://doi.org/10.1039/D0TC00388C.

(33)	Griffiths, K.; Halcovitch, N. R.; Griffin, J. M. Long-Term Solar Energy Storage under Ambient Conditions in a MOF-Based Solid–Solid Phase-Change Material. *Chem. Mater.* **2020**, *32* (23), 9925–9936. https://doi.org/10.1021/acs.chemmater.0c02708.

(34)	Hjorth Larsen, A.; Jørgen Mortensen, J.; Blomqvist, J.; Castelli, I. E.; Christensen, R.; Dułak, M.; Friis, J.; Groves, M. N.; Hammer, B.; Hargus, C.; Hermes, E. D.; Jennings, P. C.; Bjerre Jensen, P.; Kermode, J.; Kitchin, J. R.; Leonhard Kolsbjerg, E.; Kubal, J.; Kaasbjerg, K.; Lysgaard, S.; Bergmann Maronsson, J.; Maxson, T.; Olsen, T.; Pastewka, L.; Peterson, A.; Rostgaard, C.; Schiøtz, J.; Schütt, O.; Strange, M.; Thygesen, K. S.; Vegge, T.; Vilhelmsen, L.; Walter, M.; Zeng, Z.; Jacobsen, K. W. The Atomic Simulation Environment—a Python Library for Working with Atoms. *J. Phys.: Condens. Matter* **2017**, *29* (27), 273002. https://doi.org/10.1088/1361-648X/aa680e.

(35)	Blum, V.; Gehrke, R.; Hanke, F.; Havu, P.; Havu, V.; Ren, X.; Reuter, K.; Scheffler, M. Ab Initio Molecular Simulations with Numeric Atom-Centered Orbitals. *Computer Physics Communications* **2009**, *180* (11), 2175–2196. https://doi.org/10.1016/j.cpc.2009.06.022.

(36)	Knuth, F.; Carbogno, C.; Atalla, V.; Blum, V.; Scheffler, M. All-Electron Formalism for Total Energy Strain Derivatives and Stress Tensor Components for Numeric Atom-Centered Orbitals. *Computer Physics Communications* **2015**, *190*, 33–50. https://doi.org/10.1016/j.cpc.2015.01.003.

(37)	Ren, X.; Rinke, P.; Blum, V.; Wieferink, J.; Tkatchenko, A.; Sanfilippo, A.; Reuter, K.; Scheffler, M. Resolution-of-Identity Approach to Hartree–Fock, Hybrid Density Functionals, RPA, MP2 and *GW* with Numeric Atom-Centered Orbital Basis Functions. *New J. Phys.* **2012**, *14* (5), 053020. https://doi.org/10.1088/1367-2630/14/5/053020.

(38)	Yu, V. W.; Corsetti, F.; García, A.; Huhn, W. P.; Jacquelin, M.; Jia, W.; Lange, B.; Lin, L.; Lu, J.; Mi, W.; Seifitokaldani, A.; Vázquez-Mayagoitia, Á.; Yang, C.; Yang, H.; Blum, V. ELSI: A Unified Software Interface for Kohn–Sham Electronic Structure Solvers. *Computer Physics Communications* **2018**, *222*, 267–285. https://doi.org/10.1016/j.cpc.2017.09.007.

(39)	Havu, V.; Blum, V.; Havu, P.; Scheffler, M. Efficient Integration for All-Electron Electronic Structure Calculation Using Numeric Basis Functions. *Journal of Computational Physics* **2009**, *228* (22), 8367–8379. https://doi.org/10.1016/j.jcp.2009.08.008.

(40)	Ihrig, A. C.; Wieferink, J.; Zhang, I. Y.; Ropo, M.; Ren, X.; Rinke, P.; Scheffler, M.; Blum, V. Accurate Localized Resolution of Identity Approach for Linear-Scaling Hybrid Density Functionals and for Many-Body Perturbation Theory. *New J. Phys.* **2015**, *17* (9), 093020. https://doi.org/10.1088/1367-2630/17/9/093020.

(41)	Perdew, J. P.; Burke, K.; Ernzerhof, M. Generalized Gradient Approximation Made Simple. *Phys. Rev. Lett.* **1996**, *77* (18), 3865–3868. https://doi.org/10.1103/PhysRevLett.77.3865.

(42)	Tkatchenko, A.; DiStasio, R. A.; Car, R.; Scheffler, M. Accurate and Efficient Method for Many-Body van Der Waals Interactions. *Phys. Rev. Lett.* **2012**, *108* (23), 236402. https://doi.org/10.1103/PhysRevLett.108.236402.

(43)	Ambrosetti, A.; Reilly, A. M.; DiStasio, R. A.; Tkatchenko, A. Long-Range Correlation Energy Calculated from Coupled Atomic Response Functions. *The Journal of Chemical Physics* **2014**, *140* (18), 18A508. https://doi.org/10.1063/1.4865104.

(44)	Togo, A. First-Principles Phonon Calculations with Phonopy and Phono3py. *J. Phys. Soc. Jpn.* **2023**, *92* (1), 012001. https://doi.org/10.7566/JPSJ.92.012001.

(45)	Togo, A.; Chaput, L.; Tadano, T.; Tanaka, I. Implementation Strategies in Phonopy and Phono3py. *J. Phys.: Condens. Matter* **2023**, *35* (35), 353001. https://doi.org/10.1088/1361-648X/acd831.

(46)	Skelton, J. M.; Burton, L. A.; Jackson, A. J.; Oba, F.; Parker, S. C.; Walsh, A. Lattice Dynamics of the Tin Sulphides SnS_2_ , SnS and Sn_2_ S_3_ : Vibrational Spectra and Thermal Transport. *Phys. Chem. Chem. Phys.* **2017**, *19* (19), 12452–12465. https://doi.org/10.1039/C7CP01680H.

(47)	Skelton-Group/Phonopy-Spectroscopy, 2026. https://github.com/skelton-group/Phonopy-Spectroscopy (accessed 2026-01-16).

(48)	*Jmol: an open-source Java viewer for chemical structures in 3D*. https://jmol.sourceforge.net/ (accessed 2026-01-16).

(49)	Tang, Y.; Dubbeldam, D.; Tanase, S. Water–Ethanol and Methanol–Ethanol Separations Using in Situ Confined Polymer Chains in a Metal–Organic Framework. *ACS Appl. Mater. Interfaces* **2019**, *11* (44), 41383–41393. https://doi.org/10.1021/acsami.9b14367.

(50)	Kovalenko, V. I.; Akhmadiyarov, A. A.; Vandyukov, A. E.; Khamatgalimov, A. R. Experimental Vibrational Spectra and Computational Study of 1,4-Diazabicyclo[2.2.2]Octane. *Journal of Molecular Structure* **2012**, *1028*, 134–140. https://doi.org/10.1016/j.molstruc.2012.06.045.

(51)	Lugier, O.; Pokharel, U.; Castellanos, S. Impact of Synthetic Conditions on the Morphology and Crystallinity of FDMOF-1(Cu) Thin Films. *Crystal Growth & Design* **2020**, *20* (8), 5302–5309. https://doi.org/10.1021/acs.cgd.0c00529.

(52)	Baumgartner, B.; Ikigaki, K.; Okada, K.; Takahashi, M. Infrared Crystallography for Framework and Linker Orientation in Metal–Organic Framework Films. *Chem. Sci.* **2021**, *12* (27), 9298–9308. https://doi.org/10.1039/D1SC02370E.

(53)	Henschel, H.; Andersson, A. T.; Jespers, W.; Mehdi Ghahremanpour, M.; Van Der Spoel, D. Theoretical Infrared Spectra: Quantitative Similarity Measures and Force Fields. *J. Chem. Theory Comput.* **2020**, *16* (5), 3307–3315. https://doi.org/10.1021/acs.jctc.0c00126.

(54)	Fischer, J. C.; Li, C.; Hamer, S.; Heinke, L.; Herges, R.; Richards, B. S.; Howard, I. A. GIWAXS Characterization of Metal–Organic Framework Thin Films and Heterostructures: Quantifying Structure and Orientation. *Adv Materials Inter* **2023**, *10* (11), 2202259. https://doi.org/10.1002/admi.202202259.

(55)	Oesinghaus, L.; Schlipf, J.; Giesbrecht, N.; Song, L.; Hu, Y.; Bein, T.; Docampo, P.; Müller‐Buschbaum, P. Toward Tailored Film Morphologies: The Origin of Crystal Orientation in Hybrid Perovskite Thin Films. *Adv Materials Inter* **2016**, *3* (19), 1600403. https://doi.org/10.1002/admi.201600403.

(56)	Gasser, F.; Simbrunner, J.; Huck, M.; Moser, A.; Steinrück, H.-G.; Resel, R. Intensity Corrections for Grazing-Incidence X-Ray Diffraction of Thin Films Using Static Area Detectors. *J Appl Crystallogr* **2025**, *58* (1), 96–106. https://doi.org/10.1107/S1600576724010628.

(57)	Jiang, Z. *GIXSGUI* : A MATLAB Toolbox for Grazing-Incidence X-Ray Scattering Data Visualization and Reduction, and Indexing of Buried Three-Dimensional Periodic Nanostructured Films. *J Appl Crystallogr* **2015**, *48* (3), 917–926. https://doi.org/10.1107/S1600576715004434.

(58)	Yoneda, Y. Anomalous Surface Reflection of X Rays. *Phys. Rev.* **1963**, *131* (5), 2010–2013. https://doi.org/10.1103/PhysRev.131.2010.

(59)	Lafrance, C. P.; Pezolet, M.; Prud’homme, R. E. Study of the Distribution of Molecular Orientation in Highly Oriented Polyethylene by X-Ray Diffraction. *Macromolecules* **1991**, *24* (17), 4948–4956. https://doi.org/10.1021/ma00017a035.
